# Child-appealing packaged food and beverage products in Canada–Prevalence, power, and nutritional quality

**DOI:** 10.1371/journal.pone.0284350

**Published:** 2023-05-03

**Authors:** Christine Mulligan, Laura Vergeer, Monique Potvin Kent, Mary R. L’Abbé

**Affiliations:** 1 Department of Nutritional Sciences, University of Toronto, Toronto, Ontario, Canada; 2 School of Epidemiology and Public Health, Faculty of Medicine, University of Ottawa, Ottawa, Canada; Wroclaw University of Environmental and Life Sciences: Uniwersytet Przyrodniczy we Wroclawiu, POLAND

## Abstract

**Background:**

Children are frequently exposed to marketing on food packaging. This study evaluated the presence, type and power of child-appealing marketing and compared the nutritional quality of child-appealing vs. non-child-appealing Canadian packaged foods and examined the relationship between nutrient composition and marketing power.

**Methods:**

Child-relevant packaged foods (n = 5,850) were sampled from the Food Label Information Program 2017 database. The presence and power (# of techniques displayed) of child-appealing marketing were identified. Fisher’s Exact test compared the proportion of products exceeding Health Canada’s nutrient thresholds for advertising restrictions and Mann Whitney U tests compared nutrient composition between products with child- /non-child-appealing packaging. Pearson’s correlation analyzed the relationship between nutrient composition and marketing power.

**Results:**

13% (746/5850) of products displayed child-appealing marketing; the techniques used, and the power of the marketing varied ($\bar{x}$ 2.2 techniques; range: 0–11). More products with child-appealing packaging than with non-child appealing packaging exceeded Health Canada’s thresholds (98% vs. 94%; p < .001). Products with child-appealing packaging (vs. non-child-appealing) were higher in total sugars (median: 14.7 vs. 9 g/RA; p < .001) and free sugars (11.5 vs. 6.2 g/RA; p < .001), but lower in all other nutrients. There was weak overall correlation between marketing power and nutrient levels. Results varied by nutrient and food category.

**Conclusions:**

Unhealthy products with powerful child-appealing marketing displayed on package are prevalent in the food supply. Implementing marketing restrictions that protect children should be a priority.

## Introduction

Children are exposed to an exorbitant volume of marketing for foods and beverages, across a variety of marketing channels and settings [1–6]. This problem is exacerbated by the overwhelming consensus in the literature describing the poor nutritional quality of products that are marketed in a child-appealing manner, regardless of the nutritional assessment method (or nutrient profile model) used for evaluation [4, 5, 7]. Food marketing’s influence on children is a function of both their exposure to the advertising, as well as the power (or the persuasiveness) of that marketing [6, 8]. Evidence has shown that unhealthy food marketing has impacts on children’s taste preferences and consumption behaviours [4, 6, 9]. As a result, children’s diet quality suffers, which contributes to the growing global burden of childhood overweight, obesity, diabetes, and diet-related chronic disease [1, 10, 11]. Evidence from Canada has shown that children’s overall diet quality is poor, in part due to inadequate fruit and vegetable consumption paired with high intakes of highly processed, nutrient-poor foods [12–15]. The marketing of unhealthy foods to children has therefore been identified as an urgent public health concern in Canada, and globally.

Following the release of recommendations and protocols aimed at helping countries restrict and monitor child-appealing marketing from several prominent health organizations, many countries have responded by implementing (or beginning to develop) voluntary or mandatory policies to restrict unhealthy marketing practices to children [1, 6, 8, 16–19]. In Canada Bill S-228, *The Child Health Protection Act*, was proposed in 2016 aiming to federally mandate the restriction of marketing unhealthy foods to children under 13 [20]. However, this bill was never passed, which is unfortunate given emerging evidence which indicates that mandatory restrictions are in fact effective methods to reduce children’s exposure to marketing for foods that are high in nutrients of public health concern (e.g., sodium, sugars, saturated fats) [21–24].

To support and inform the widespread development and implementation of marketing restrictions, continued monitoring of child-appealing marketing is necessary [8]. An important question in this field, however, is how to define and measure whether marketing is “child-appealing”. The current lack of standardization in terms of definitions and methodologies for evaluating child-appeal is concerning given the potential for heterogeneity—and therefore incomparability—in research and policy outcomes [25–27].

Product packaging has been identified as a top source of children’s exposure to marketing for unhealthy foods and is a platform often neglected by restrictions and regulations, presenting a dangerous loophole for food and beverage manufacturers to exploit [26–28]. Such a loophole exists in Canada, where food and beverage marketing is still mostly self-regulated by the Canadian Children’s Food and Beverage Advertising Initiative which restricts less healthy advertising to children under age 12 in many media and settings, with the exclusion of product packaging [29]. Previous work examining marketing on product packaging has tracked a range (in both the type and number) of marketing techniques, making it challenging to compare across studies, but techniques such as the use of characters, cross-promotions, games, toys, and child-appealing graphic designs (e.g., unusual colors, shapes) appear to be displayed frequently on package [25, 30–33]. There has also been little empirical evaluation of marketing power to date [25, 27], despite power being an important construct to consider according to the World Health Organization [6]. Recently, a novel methodology was published to broadly and consistently measure the presence, type, and power (or persuasiveness) of child-appealing food marketing on packaging [26], but this methodology has yet to be used to evaluate a large sample of products. Moreover, how the power of child-appealing marketing relates to the healthfulness (or lack thereof) and nutrient levels of food and beverage products has yet to be elucidated on any marketing platform. Additionally, despite the evidence of Canadian children’s poor diet quality which may be putting them at increased risk for micronutrient deficiency, as well as evidence to suggest that products marketing in a child-appealing manner could promote nutrient adequacy and in fact be strong sources of nutrients to encourage within the diet (e.g., calcium, fibre), research in this field has largely neglected evaluating the levels of these more positive nutrients and has rather focused primarily on the negative dietary contributions of products children are frequently exposed to [34–38].

The objectives of this study were therefore to a) measure the presence, type, and power of child-appealing marketing on product packaging using a newly developed methodology and b) evaluate the nutritional quality of child-appealing (vs. non-child-appealing) products and c) investigate the relationship between nutritional quality and marketing power. It is hypothesized that products with child-appealing packaging are of poorer nutritional quality than products with non-child-appealing packaging, and that marketing power is negatively correlated with nutritional quality (i.e., more powerfully marketed products are less healthy).

## Methods

### Food label information program

This study was a cross sectional analysis of the Food Label Information Program (FLIP) 2017 database, developed and maintained by the University of Toronto, full details for which can be found elsewhere [39]. Briefly, FLIP 2017 contains package information for over 17,000 branded packaged food and beverage products from three top Canadian food retailers. Relevant to the present study, FLIP 2017 contains all data from a product’s Nutrition Facts table (NFt) and ingredients list, as well as photos of all sides of the product packaging, collected in-store. Nutrient information in FLIP is available “as sold” (i.e., as visible to the consumer on the NFt at the time of purchase) and “as prepared” (i.e., derived based on preparation instructions listed on the product), where relevant. All products in FLIP are categorized according to Health Canada’s Table of Reference Amounts for Foods (TRA) major food categories (e.g., bakery products) and subcategories (e.g., cookies) [40].

The analytic sample for this study was derived by selecting a subset of TRA subcategories that were deemed to be of top priority for analyses of child-appealing marketing, based on previous evaluations in FLIP 2013 [7, 41]. Subcategories were ranked based on the number of products that were categorized as displaying child-appealing packaging in FLIP 2013 and the subcategories making up the top 85% of child-appealing packages were selected. Any additional subcategories with >10% child-appealing packages were added to the sample. The final analytic sample of n = 5,850 products from FLIP 2017 contained subcategories that captured 89% of products displaying child-appealing marketing on packaging in FLIP 2013. Included subcategories are listed in **S1 Table in S1 File**.

### Child-appealing packaging (CAP) coding tool

The CAP coding tool was developed to measure child-appealing marketing on food packaging, based on the marketing techniques that are displayed. A previous publication details the development and pilot mixed-methods validation of the CAP tool [26]. Briefly, the CAP tool identifies a series of core (i.e., techniques that could independently make a package appealing to children) and broad marketing techniques (i.e., marketing techniques that would not on their own cause a package to be child-appealing, but that increase the overall persuasiveness of the marketing message). **Table 1** describes the marketing techniques coded by the CAP tool. Based on the display of these core and broad techniques, the CAP tool then evaluates 1) the presence of child-appealing marketing (i.e., if one or more core techniques were displayed), 2) the type of marketing (i.e., which individual techniques were being displayed) and 3) the power of the marketing—the marketing power score (MPS)–determined by the total number of marketing techniques that were displayed on the package [26]. The CAP coding tool was used to evaluate all products in the sample by identifying the display of marketing techniques on all sides of the packaging.

**Table 1:** Description of marketing techniques coded by the child-appealing packaging tool.

**CORE MARKETING TECHNIQUES**		
**Technique**	**Definition**	**Examples**
**Child-appealing visual/graphical design of package**	Intense colors, patterns or visual designs on the packaging or design themes related to fantasy, adventure, magic, sports, etc. that are clearly appealing to children. This can also include the presence of cartoon characters, children, animals, etc. that **are not** branded or licensed characters, celebrities, or tie-ins to child-appealing media. *Note*: *this can include child-appealing lettering*, *if it is enough on its own for the product to be considered “child-appealing”*, *otherwise code lettering under broad techniques*.	○ Space-themed visual design ○ Rainbow packaging ○ Chalkboard-style lettering ○ Cartoon pictures of fictional sports players ○ Cartoon drawings of animals ○ Cartoon kids or families
**Unconventional shape of the product, featured on the package**	The product featured on the packaging has a shape that is unconventional or unusual for that type of product. E.g. if crackers have a shape other than their usual square or round shape. *Note*: *In the case of clear plastic containers where the product is visible through the package*, *this counts as the shape being visible*.	○ Animal shaped crackers ○ Alphabet shaped pasta ○ Character, fruit or animal shaped gummies
**Unconventional flavour of the product, featured on the package**	The product featured on the package has a flavour that is unconventional or unusual for that type of product, or a flavour that is not a ‘real’ or ‘discernable’ flavour. *Note*: *this could include the presentation of the flavour in a ‘negative’ way that may appeal to children; e*.*g*., *tastes crazy*, *weird*, *sour*, *whacky*	○ Tropical Storm Flavour ○ Cheddarific ○ Secret Flavour ○ Chocolate Mud flavour ○ Cool Cucumber flavour ○ Raspberry Kiwi Karma
**Unconventional colour of the product, featured on the package**	The product featured on the package has a colour that is unconventional or unusual for that type of product. E.g. if crackers are coloured rather than their usual plain/brown colour. *Note*: *In the case of clear plastic containers where the product is visible through the package*, *this counts as the color being visible*.	○ Rainbow crackers ○ Purple Ketchup ○ Colour changing drink powder ○ Rainbow fruit roll ups (instead of just red, for example) *Note*: *multi-colored candies would NOT be unusual*, *unless they are described in a more ‘fun’ or child-appealing way*.
**Games or activities on package**	Presence of games or activities on the package.	○ Connect the dots ○ Mazes ○ “Count how many snowmen”
**Presence of branded characters or spokespersons**	Presence of company- or brand-owned characters.	○ Tony the Tiger ○ Toucan Sam ○ Cap’n Crunch ○ Kraft Bears ○ Pillsbury Doughboy
**Presence of Licensed Characters**	Presence of characters from TV shows, movies, books, etc., that may appeal to children. Note: human actors, if presented as the character are included here (e.g., Miley Cyrus as Hannah Montana), if portrayed as themselves, include under “Presence of Celebrities” (e.g., Miley Cyrus advertised as Miley Cyrus).	○ Dora the Explorer ○ Batman ○ Hannah Montana ○ Star Wars characters
**Presence of celebrities**	Presence of actors, athletes, musicians, other public figures that may appeal to children	○ Derek Jeter ○ Miley Cyrus
**Other child-appealing tie-ins**	Other movie/sports/TV show etc. tie-ins that are appealing to children are advertised on the package aside from one of the types of characters or celebrities described above. *Note*: *these may appear in addition to the presence of any characters described above*	○ NHL tie-ins that feature an ice-rink or hockey equipment with/without a specific player. ○ Harry Potter tie-in where Hogwarts is presented with/without a character.
**Coupons, contests, prizes, or giveaways, specifically appealing to children**	Coupons, contests or prizes or giveaways inside the package or to be redeemed later. *Note*: *contests or giveaways must be for child-appealing prizes (unlike*, *for e*.*g*., *a Patio Furniture set)*	○ Enter to win tickets to a child-appealing movie ○ Coupon for free yogurt tubes ○ Toys inside package ○ Stickers inside package
**Appeals to fun or cool**	Product packaging makes appeals to the product being fun or funny, having fun while eating the product, being happy, enjoyment, humour, coolness, being cool, etc. *Note*: *this includes “fun” packaging (i*.*e*., *Packaging that is designed in a way to promote “fun” during eating*, *or makes eating an “activity”)* *Note*: *this could be as part of the product name (e*.*g*., *“Fun Dip”*, *“Kool Kreatures”)*, *if it is clearly “fun/cool” and appealing to children*	○ “Have more fun with” ○ “Feel the bubbles melt” ○ “Try our crazy/cool new flavors” ○ “Smiles included” ○ Display of children having fun, being happy, enjoying the product ○ Yogurt Tubes ○ Dunkaroos ○ Chips Ahoy! ○ Processed cheese with dipping breadsticks (if “dipping” is promoted as an activity)
**Promotion of websites, social media, rewards programs, specifically appealing to children**	Product packaging promotes product/brand/company website, child-specific or games-based brand website, social media, or opportunities to “join”, “become a member”, redeem points, and collect rewards or to connect or share with others in a manner that is evidently child-appealing	○ “Find more games on [website]” ○ References to “kids club” or similar
**BROAD MARKETING TECHNIQUES**		
**Technique**	**Definition**	**Examples**
**Interesting font or lettering**	Presence of product name or description (e.g., product flavour) written or designed in a colorful, creative, or interesting font that is not on its own enough to make the package “child-appealing”, but may contribute to the overall power of the marketing. *Note*: *this broad technique exists due to the difficult nature of identifying child-appealing lettering*, *and since often products will use bubbly or colorful fonts*, *but this alone is not always enough to consider a product child-directed*. *Note*: *if the lettering is enough to make the product child-appealing*, *count under technique #1 child-appealing visual/graphic design*	○ Aero bar bubble lettering ○ Corn Pops lettering ○ Cheetos lettering
**Interesting or unconventional product name**	Unconventional product name (e.g., strange spelling, rhyming, and alliteration) that may be interesting to children and build marketing power. *Note*: *if not counted as part of a core technique (e*.*g*., *appeals to fun/cool or visual/graphic design of the package) and* ***not enough*** *to make the product child-appealing on its own*.	○ Frooty Hoops ○ Juicy Jels ○ Wagon Wheels ○ “Eat the middle first”
**Presence of a logo/image not specifically appealing to children**	Presence of a product/brand logo or a cartoon or image that is not specifically appealing to children. This could include pictures of families or children consuming the product (if not in a child-appealing way)	○ The man with a moustache in the Pringles logo ○ Quaker Oats man in the logo ○ Realistic cows on cheese products Picture of a child drinking milk on the side of a soy milk package
**Promotion of convenient packaging**	Specific promotion of the product being packaged in a convenient or easy way. *Note*: *if the packaging is promoted as “fun” or as an activity count under “appeals to fun/cool”Note*: *this does not include single serve packaging (e*.*g*., *juice boxes or crackers and cheese packs) without specific promotion of their convenience*.	○ “Perfect for on-the-go snacking.” ○ “Great for packing in lunches” ○ “Contains 6 easy servings”
**Appeals to taste or texture**	Product packaging makes appeals to the flavour taste, or texture, of the product, in a way that is not specifically appealing to children. *Note: this includes if the appeal to taste/texture is part of the product name/brand or product description.	○ “New look, same great taste” ○ “You’ll love it” ○ “Deep n’ Delicious!” ○ “Tastes like mama made it” ○ Promotion of textures (e.g., crunchy, smooth…) ○ Improved recipe!
**Appeals to health or nutrition**	Product packaging makes appeals to the healthfulness or nutritional quality of the product, its ability to promote growth, strength, or physical activity. Product packaging displays “healthy foods” alongside the product. *Note*: *includes health and nutrition claims/symbols*, *as well as organic or natural claims/symbols*	○ “Helps them grow strong” ○ “Part of a healthy breakfast” ○ Fruit featured beside the product on pack (e.g. bowl of strawberries beside cereal) ○ Source of 5 whole grains ○ Made with 100%. . . ○ Promotion of ‘real’, ‘pure’, ‘natural’ etc. ○ Gluten free or vegetarian symbol ○ Peanut free symbol
**Appeals to other product benefits**	Product packaging makes appeals to other product benefits aside from health/taste/fun. For example, value, quickness, easy preparation, sustainability, philanthropy, etc. *Note*: *this does not include small statements (often on the bottom of the package) that the package was made from recycled materials or is recyclable*.	○ “Quick and easy” ○ “Ready in 5 minutes” ○ “Ready to bake” ○ Proceeds go to X organization ○ B Certified Corporations ○ Promotion of local ingredients ○ Promotion of “made in Canada” ○ Value pack/family size/club pack
**Recipes**	Product packaging displays recipes that can be made using the product *Note: does not include standard cooking instructions for products that require preparation (e.g., how to cook a frozen pizza)	○ Rice crispy squares ○ Bran muffins ○ Low calorie smoothies ○ “Chef’s Tip”
**Promotion of websites, social media, rewards programs, not specifically appealing to children**	Product packaging promotes product/brand/company website, social media, or opportunities to “join”, “become a member”, redeem points, and collect rewards or to connect or share with others, in a way that is not specifically child-appealing *Note*: *does not include link/QR code to company/manufacturer website included as part of contact information on package*	○ Social Media links ○ Links to recipe websites ○ Links to “create the next flavour of chips” ○ QR codes (if promoted in a special way)
**Coupons, contests, or giveaways, not specifically appealing to children**	Coupons, contests or giveaways to be entered or redeemed later that are not specifically appealing to children.	○ Tote bags ○ Access to a free weight loss plan ○ Patio furniture set

To ensure consistency and reliability in coding, initially, two researchers (C.M. and L.V.) independently coded a random 5% of the sample, serving as a training phase prior to the rest of the data being coded. Inter-rater reliability checks found 93.2% raw agreement in coding whether products were child appealing. Where there were consistent discrepancies or uncertainties around the coding of specific marketing techniques, researchers reached a consensus on how to code such products, and minor modifications and/or clarifications to the CAP tool were made to facilitate the consistent coding for the remainder of the sample. The updated version of the CAP tool that was used in this study can be found in **S1 Appendix in S1 File**. A single researcher (C.M.) coded the rest of the sample according to the decisions made during the initial training/validation phase. Once the full coding was complete, a second researcher (L.V.) checked all products where there was any uncertainty (n = 153 products) as well as a random 2.5% sample (n = 194 products), among which raw agreement across all marketing techniques was 99.6%, resulting in a Cohen’s Kappa Statistic of 0.98, signifying near perfect. All discrepancies were resolved through consensus between researchers.

### Analysis

#### Analysis of marketing displayed on package

The number and proportion of products that would be considered child-appealing (i.e., displaying 1 or more core marketing techniques listed in Table 1), the number and proportion of products displaying each individual marketing technique, and the mean (and standard deviation), median and range (min-max) of core techniques displayed, broad techniques displayed, and MPS (i.e., the total number of core and broad techniques displayed) were calculated overall and by food category.

#### Assessment of nutritional quality

All products were evaluated under the nutrient thresholds proposed by Health Canada as part of their regulatory proposal to accompany Bill S-228 [42]. Briefly, Health Canada outlined thresholds for three critical nutrients (i.e., sodium, total sugars, and saturated fat) at approximately 5% of the Daily Value for each [41–43]. Detailed methodology on the application of the Health Canada thresholds in the FLIP database have been published elsewhere [41]. According to the proposal, if a product exceeds one or more of the nutrient thresholds, it would not be permitted to be marketed to children. The number of products that would or would not be permitted to be marketed to children was calculated and the difference between the proportion of permitted products between products with child-appealing and non-child-appealing packaging was assessed using Fisher’s Exact test due to low expected values in many food categories. Student’s T test was used to compare the mean MPS between permitted and restricted products, overall and within child-appealing and non-child-appealing packages. The proportion of products exceeding each individual threshold, as well as the mean MPS per number of thresholds exceeded (i.e., 0, 1, 2, or 3) was analyzed overall and per food category.

The median number (and interquartile range) of energy (i.e., kcal), total fats, saturated fats, trans fats, total sugars, free sugars, sodium, protein, fibre, calcium, and iron were calculated per TRA reference amount (RA). If a product’s package stated that preparation was required, “as prepared” nutritional quality was assessed, otherwise products were assessed “as sold”. This is consistent with how products were evaluated under Health Canada’s nutrient thresholds [41]. Nutrient levels in products with child-appealing and non-child-appealing packaging were compared overall and within food categories using Mann-Whitney U tests due to the non-parametric nature of nutrition composition data. Statistical tests were not performed in categories where the number of products with either child- or non-child-appealing packaging was < 10 (i.e., Drinkable Yogurt, Hot Cocoa, Meats, Shakes, and Toaster Pastries) due to insufficient statistical power. Spearman correlation analyses assessed the relationship between marketing power and nutrient composition for all nutrients, overall and by food category. Values of Spearman’s *r*_*s*_ were interpreted as follows: 0.00 as “zero”, 0.01–0.30 as “weak”, 0.31–0.60 as “moderate”, 0.61–0.99 as “strong”, and 1.00 as “perfect” [44]. For all analyses, p values < .05 were considered statistically significant.

## Results

### Analysis of child-appealing marketing on a sample of Canadian food packages

Overall, 12.8% (n = 747/5850) of products had child-appealing packaging **(Table 2)**. The categories with the highest proportion of products displaying child-appealing marketing on their packages were Toaster Pastries (100%, n = 11/11), Cereals (53.4%, n = 47/88), and Crackers (42.4%, n = 28/66), with moderate levels in Candy (26.4%, n = 151/573), Fruit Sauce (26.2%, n = 17/65), Nut Butters (22.8%, n = 23/101), Syrups/Spreads (20.7%, n = 19/22), and Ice Cream (19.8%. n = 99/499). There were also several categories with a high absolute number of child-appealing packages: Candy (n = 151), Ice cream (n = 99), Cookies (n = 84), Meals (n = 66) and Juice (n = 65). Categories with the lowest proportion of products with child-appealing packaging were Cheese (1.9%, n = 11/569), Hot Cocoa (3.1%, n = 1/32), and Meats (3.3%, n = 7/214).

**Table 2:** Number (n) and proportion (%) of products with child-appealing packaging overall and by food category.

Food Category	Total n	Child-appealing packaging		Non-child-appealing packaging	
		n	%	n	%
Cakes	155	10	6.5	145	93.5
Candy	573	151	26.4	422	73.6
Cereal	88	47	53.4	41	46.6
Cheese	569	11	1.9	558	98.1
Cookies	523	84	16.1	439	83.9
Crackers	66	28	42.4	38	57.6
Drinkable Yogurt	60	6	10.0	54	90.0
Fruit Sauce	65	17	26.2	48	73.8
Grain Bars	199	30	15.1	169	84.9
Hot Cocoa	32	1	3.1	31	96.9
Ice Cream	499	99	19.8	400	80.2
Juice	608	56	9.2	552	90.8
Meals	1012	66	6.5	946	93.5
Meats	214	7	3.3	207	96.7
Milk	201	18	9.0	183	91.0
Nut Butter	101	23	22.8	78	77.2
Pudding	180	17	9.4	163	90.6
Shakes	25	7	28.0	18	72.0
Snacks	577	38	6.6	539	93.4
Syrups/Spreads	92	19	20.7	73	79.3
Toaster Pastries	11	11	100.0	0	0.0
**OVERALL**	**5850**	**746**	**12.8**	**5104**	**87.2**

The display of core and broad marketing techniques varied across food categories. Overall, 12.8% of products displayed at least one core marketing technique, meaning that their packaging was considered “child-appealing”, with the most frequently displayed techniques being “child-appealing visual/graphical design” (10.0% of products), “appeals to fun or cool” (3.3%) and “presence of branded characters or spokespersons” (3.2%) **(S2 Table in S1 File)**. Overall, 90.8% of products displayed one or more broad marketing technique, with “appeals to health or nutrition” (71.2%), “appeals to taste or texture” (44.5) and “websites/social media” (30.3%) being most common.

The overall mean ($\bar{x}$ (SD)) number of core techniques displayed per product was 0.2 (0.7) techniques with a median of 0 techniques and a range (min-max) of 0–7 techniques. The categories the highest mean number of core techniques displayed were Toaster Pastries ($\bar{x}$ 1.7 (0.6)), Crackers ($\bar{x}$ 1.5 (2.0)) and Cereal ($\bar{x}$ 1.3 (1.6)) **(Table 3)**. The overall mean number of broad techniques displayed per product was 2.0 (1.2) techniques, with a median of 2 techniques and a range of 0–6 techniques. The categories with the highest mean number of broad techniques displayed were Cereal ($\bar{x}$ 3.2 (1.0)), Milk ($\bar{x}$ 2.9 (0.9)), and Ice Cream ($\bar{x}$ 2.6 (1.9)). The overall mean MPS was 2.2 (1.5), with a median of 2 and a range of 0–11 techniques. The categories with the highest mean MPS were Cereal ($\bar{x}$ 4.5 (2.0)), Crackers ($\bar{x}$ 3.8 (2.5)), and Toaster Pastries ($\bar{x}$ 3.1 (1.2)).

**Table 3:** Distribution of core and broad marketing technique display and marketing power scores, overall and per food category.

	Number of core marketing techniques displayed^1^			Number of broad marketing techniques displayed^1^			Marketing Power Score^2^		
Food Category	Mean (SD)	Median	Range (Min-Max)	Mean (SD)	Median	Range (Min-Max)	Mean (SD)	Median	Range (Min-Max)
Cakes	0.1 (0.3)	0	0–2	1.2 (1.3)	1	0–4	1.3 (1.3)	1	0–4
Candy	0.5 (0.9)	0	0–5	1.7 (1.2)	2	0–5	2.2 (1.5)	2	0–8
Cereal	1.3 (1.6)	1	0–7	3.2 (1)	3	1–5	4.5 (2)	4	1–11
Cheese	0 (0.4)	0	0–4	1.5 (1.1)	1	0–6	1.6 (1.3)	1	0–10
Cookies	0.4 (1)	0	0–5	2.2 (1.3)	2	0–6	2.5 (1.8)	2	0–8
Crackers	1.5 (2)	0	0–6	2.3 (0.9)	2	0–5	3.8 (2.5)	3	0–9
Drinkable Yogurt	0.2 (0.7)	0	0–3	2.6 (1.1)	2	1–5	2.8 (1.5)	2	1–7
Fruit Sauce	0.4 (0.8)	0	0–3	2.4 (1.6)	2	0–6	2.8 (2.2)	2	0–9
Grain Bars	0.4 (1)	0	0–4	2.5 (1.1)	2	0–5	2.9 (1.7)	2	0–9
Hot Cocoa	0 (0.2)	0	0–1	2.3 (1.1)	2	0–4	2.4 (1.1)	2	0–4
Ice Cream	0.4 (0.8)	0	0–4	2.6 (1.9)	3	1–5	2.9 (1.1)	3	1–7
Juice	0.2 (0.6)	0	0–3	1.7 (1)	1	0–5	1.8 (1.1)	2	0–6
Meals	0.1 (0.5)	0	0–4	1.8 (1.2)	2	0–6	2 (1.4)	2	0–9
Meats	0.1 (0.3)	0	0–2	1.8 (0.8)	2	0–4	1.9 (0.9)	2	0–5
Milk	0.1 (0.3)	0	0–2	2.9 (0.9)	3	0–5	3 (0.9)	3	0–5
Nut Butter	0.3 (0.6)	0	0–2	1.9 (1)	2	0–6	2.2 (1.3)	2	0–6
Pudding	0.1 (0.3)	0	0–1	1.5 (1.1)	1	0–4	1.6 (1.2)	1	0–4
Shakes	0.4 (0.8)	0	0–3	1.9 (1.1)	1	1–4	2.3 (1.5)	2	1–6
Snacks	0.1 (0.5)	0	0–3	2.4 (1.2)	2	0–5	2.5 (1.2)	3	0–6
Syrups/Spreads	0.3 (0.7)	0	0–3	1.5 (1.2)	1	0–5	1.8 (1.5)	1	0–7
Toaster Pastries	1.7 (0.6)	2	1–3	1.4 (0.5)	1	1–2	3.1 (0.3)	3	3–4
**OVERALL**	**0.2 (0.7)**	**0**	**0–7**	**2 (1.2)**	**2**	**0–6**	**2.2 (1.5)**	**2**	**0–11**

^1^Core and broad marketing techniques, as defined by the child-appealing packaging (CAP) coding tool (Appendix A)

^2^Marketing power score = the total number of core and broad techniques displayed on the package

### Comparison of nutritional quality between products with child-appealing and non-child-appealing packaging using Health Canada’s nutrient thresholds for advertising restrictions

There were significantly more products with child-appealing packaging that would be restricted from being advertised to children compared to products non-child-appealing packaging (97.9% vs. 94.2%, respectively, Fisher p < .01) **(Table 4)**. More than 82% of products with child-appealing packaging in all food categories would be restricted from being advertised to children.

**Table 4:** Comparison of the proportion of products that would be permitted and restricted from advertising to children between products with child-appealing and non-child-appealing packaging, according to Health Canada’s proposed nutrient thresholds for advertising restrictions^1^.

	Child-appealing packaging					Non-child-appealing packaging					
Food category	n	Permitted for advertising to children		Restricted from advertising to children		n	Permitted for advertising to children		Restricted from advertising to children		Fisher p value
		n	%	n	%		n	%	n	%	
Cakes	10	0	0.0	10	100.0	145	0	0.0	145	100.0	NA
Candy	151	0	0.0	151	100.0	405	4	1.0	418	103.2	0.58
Cereal	47	0	0.0	47	100.0	41	2	4.9	39	95.1	0.21
Cheese	11	0	0.0	11	100.0	558	5	0.9	553	99.1	1
Cookies	84	2	2.4	82	97.6	439	3	0.7	436	99.3	0.18
Crackers	28	0	0.0	28	100.0	38	0	0.0	38	100.0	NA
Drinkable Yogurt	6	0	0.0	6	100.0	54	8	14.8	46	85.2	0.58
Fruit Sauce	17	0	0.0	17	100.0	48	0	0.0	48	100.0	NA
Grain Bars	30	2	6.7	28	93.3	169	4	2.4	165	97.6	0.22
Hot Cocoa	1	0	0.0	1	100.0	31	0	0.0	31	100.0	NA
Ice Cream	99	0	0.0	99	100.0	400	3	0.0	397	1.0	1
Juice	56	0	0.0	56	100.0	552	13	2.4	539	97.6	0.62
Meals	66	0	0.0	66	100.0	946	21	2.2	925	97.8	0.39
Meats	7	0	0.0	7	100.0	207	23	11.1	184	88.9	1
Milk	18	1	5.6	17	94.4	183	92	50.3	91	49.7	<0.001
Nut Butter	23	4	17.4	19	82.6	78	45	57.7	33	42.3	<0.001
Pudding	17	2	11.8	15	88.2	163	13	8.0	150	92.0	0.64
Shakes	7	0	0.0	7	100.0	18	0	0.0	18	100.0	NA
Snacks	38	5	13.2	33	86.8	539	57	10.6	482	89.4	0.59
Syrups/Spreads	19	0	0.0	19	100.0	90	2	2.2	71	78.9	1
Toaster Pastries	11	0	0.0	11	100.0	0	NA	NA	NA	NA	NA
**OVERALL**	**746**	**16**	**2.1**	**730**	**97.9**	**5104**	**295**	**5.8**	**4809**	**94.2**	**<0.001**

^1^Mulligan et al. (2020). Evaluating the Canadian packaged food supply using Health Canada’s proposed nutrient criteria for restricting food and beverage marketing to children. *IJERPH*, 17(4), 1250^.^

In the overall sample, there was no difference in mean MPS between products that would be permitted ($\bar{x}$ 2.3) and restricted ($\bar{x}$ 2.2) from being advertised (t: 1.6, p-value = 0.12) **(S3 Table in S1 File)**. Within products child-appealing packaging,permitted products had a lower mean MPS than restricted products ($\bar{x}$ 3.6 vs. 4.2, respectively, T: 2.2, p-value: 0.04). Within products non-child-appealing packaging, permitted products had a higher mean MPS than restricted products ($\bar{x}$ 2.3 vs. 1.9, respectively, t: 4.9, p<0.001).

The proportion of products exceeding each individual Health Canada nutrient threshold varied by food category. Within products with child-appealing packaging overall, 79.6% exceeded the sugars threshold, 42.2% exceeded the sodium threshold, and 27.2% exceeded the saturated fat threshold; whereas within products with non-child-appealing packaging, 53.6% exceeded the sugars threshold, 54.0% exceeded the sodium threshold, and 32.4% exceeded the saturated fat threshold **(S4 Table in S1 File)**. The thresholds exceeded also varied by product category. No trends were observed in the descriptive analysis of mean MPS and the number of thresholds that a product exceeded **(S5 Table in S1 File)**.

### Comparison of nutritional composition between products with child-appealing and non-child-appealing packaging

Overall, products with child-appealing packaging had lower energy than products with non-child-appealing packaging (Median ±IQR: 139.6± 78.9 kcal/RA vs. 161.5± 140.9 kcal/RA; p < .001) **Table 5**. For other ‘negative’ nutrients, products with child-appealing packaging were lower in sodium (81.3± 154.7 mg/RA vs.132.7± 298.8 mg/RA; p < .001), total fat (4± 7.9 g/RA vs. 7.7± 10 g/RA; p < .001), saturated fats (1.2± 3.2 g/RA vs. 2.4± 5.4 g/RA; p < .001), and trans fats (0± 0.1 g/RA vs. 0± 0.2 g/RA; p < .001) than products non-child-appealing packaging. However, products with child-appealing packaging had higher levels of total sugars (14.7± 3 g/RA vs. 9± 16.9 g/RA; p < .001) and free sugars (11.5± 13.5 g/RA vs. 6.2± 16 g/RA; p < .001) overall, compared to products with non-child-appealing packaging.

**Table 5:** Comparison of nutrient composition between products with child-appealing and non-child-appealing packaging, overall and per food category.

**Food Category**	**Total** ^**1**^	**Child-appealing packaging**	**Non-child-appealing packaging**	**Nutrient composition per TRA Reference Amount** ^**2**^					
				**Energy (Kcal)**			**Sodium (mg)**		
				**Median (IQR)**		**p value** ^**3**^	**Median (IQR)**		**p value** ^**3**^
	**n**	**n**	**n**	**Child-appealing**	**Non-child-appealing**		**Child-appealing**	**Non-child-appealing**	
Cakes	155	10	145	336.9 (40.4)	307.7 (69.3)	0.05	255.8 (109.3)	225 (114.3)	0.37
Candy	573	151	422	140 (21)	204.9 (41.3)	< .001	25 (35)	26.1 (33.3)	0.80
Cereal	88	47	41	117.9 (9.4)	112.5 (10)	0.18	150 (60.3)	150 (70.3)	0.91
Cheese	569	11	558	85.7 (4)	109.5 (30)	< .001	228.6 (28.6)	210 (50)	0.04
Cookies	523	84	439	137.1 (18.4)	144.8 (15)	< .001	85.7 (40.7)	84 (48)	0.07
Crackers	66	28	38	135 (15)	132 (23.7)	0.02	240 (54.1)	225 (69.1)	0.61
Drinkable Yogurt	60	6	54	101.1 (15.2)	131.6 (21.8)	NA	70.8 (7.6)	82.7 (22.3)	NA
Fruit Sauce	65	17	48	73.3 (0)	57.3 (29)	0.01	0 (6.1)	0 (9.7)	0.92
Grain Bars	199	30	169	138.5 (47.9)	140 (48.7)	0.42	89.4 (41.9)	75.7 (53.8)	0.02
Hot Cocoa	32	1	31	157.1 (0)	157.1 (75.7)	NA	50 (0)	164.3 (76.9)	NA
Ice Cream	499	99	400	180.5 (110.0)	210.6 (105.3)	< .001	70.8 (77.2)	75.1 (60.2)	0.34
Juice	608	56	552	100 (60)	120 (25.5)	< .001	20 (10.8)	15 (20)	0.30
Meals	1012	66	946	462.3 (210.6)	389.1 (144)	< .001	874.9 (243.9)	807.7 (328.3)	< .001
Meats	214	7	207	152 (54.2)	193 (113)	NA	477.9 (122)	324.7 (159.1)	NA
Milk	201	18	183	160 (37.2)	100 (68.8)	< .001	175 (40)	115 (35)	< .001
Nut Butter	101	23	78	90 (7.8)	90 (3.8)	0.01	46.9 (43.6)	15.8 (45)	< .001
Pudding	180	17	163	144.4 (39.4)	126.6 (71.8)	0.06	164.1 (98.5)	97.9 (114.7)	0.13
Shakes	25	7	18	211.9 (15)	153.8 (75.8)	NA	169.4 (42.7)	153.8 (60.8)	NA
Snacks	577	38	539	255 (50)	250 (32.5)	0.30	344.2 (150.1)	290 (182.7)	0.07
Syrups/Spreads	92	19	73	60 (42.6)	60 (53.5)	0.60	0 (22.8)	0 (10)	0.90
Toaster Pastries	11	11	0	183.3 (31.2)	NA	NA	183.3 (22)	NA	NA
**OVERALL**	**5850**	**746**	**5104**	**139.6 (78.9)**	**161.5 (140.9)**	**< .001**	**81.3 (154.7)**	**132.7 (298.8)**	**< .001**
**Food Category**	Nutrient composition per TRA Reference Amount^2^								
	**Total Fats (g)**			**Saturated Fats (g)**			**Trans Fats (g)**		
	**Median (IQR)**		**p value** ^**3**^	**Median (IQR)**		**p value** ^**3**^	**Median (IQR)**		**p value** ^**3**^
	**Child-appealing**	**Non-child-appealing**		**Child-appealing**	**Non-child-appealing**		**Child-appealing**	**Non-child-appealing**	
Cakes	13.5 (3.2)	16.8 (5.7)	0.03	6.1 (5.5)	6.9 (5)	0.62	0.2 (0.1)	0.2 (0.3)	0.20
Candy	0 (1.9)	12.1 (6.7)	< .001	0 (1.5)	7 (4)	< .001	0 (0)	0 (0.1)	< .001
Cereal	1 (1.1)	1 (1.1)	0.94	0 (0.3)	0 (0.2)	0.36	0 (0)	0 (0)	1.00
Cheese	5.7 (1.2)	8 (3.6)	0.002	3.6 (0.8)	5 (2)	0.001	0.3 (0.1)	0.3 (0.1)	0.17
Cookies	5 (2.9)	6.8 (2.6)	< .001	2 (2.2)	3 (2.5)	< .001	0 (0)	0 (0.1)	0.16
Crackers	5.8 (1.1)	4.8 (2.9)	0.06	1 (0.9)	0.6 (0.8)	0.005	0 (0)	0 (0)	0.20
Drinkable Yogurt	2 (0.8)	3 (1.9)	NA	1 (1.5)	1.9 (1.3)	NA	0 (0)	0 (0.1)	NA
Fruit Sauce	0 (0.5)	0 (0)	< .001	0 (0.1)	0 (0)	< .001	0 (0)	0 (0)	1.00
Grain Bars	3.6 (1.6)	4.6 (2.5)	0.002	1.2 (0.9)	1.3 (1.8)	0.45	0 (0)	0 (0)	0.26
Hot Cocoa	2.9 (0)	3 (2.1)	NA	2.1 (0)	2.5 (2)	NA	0 (0)	0 (0)	NA
Ice Cream	7.5 (6.9)	10.5 (10.3)	< .001	5.3 (5.4)	6 (6.5)	< .001	0.2 (0.3)	0.2 (0.3)	0.19
Juice	0 (0)	0 (0)	0.03	0 (0)	0 (0)	0.21	0 (0)	0 (0)	NA
Meals	14.1 (16.2)	12.2 (10.8)	0.08	5 (5.9)	4.1 (5.4)	0.03	0.1 (0.3)	0.1 (0.3)	0.25
Meats	7 (2.7)	11.3 (11.6)	NA	1.5 (0.9)	3.1 (6.7)	NA	0 (0)	0.1 (0.7)	NA
Milk	2.5 (0.4)	3 (2.5)	0.06	1.5 (0.5)	0.5 (2.7)	0.06	0 (0)	0 (0)	0.68
Nut Butter	7 (1.2)	7.5 (1)	0.10	1.2 (0.5)	1 (0.5)	0.36	0 (0)	0 (0)	NA
Pudding	3.9 (1.3)	2.1 (3.3)	0.002	1.3 (0.7)	1.2 (1.9)	0.50	0 (0)	0 (0)	0.10
Shakes	5.6 (1.4)	2.2 (2.3)	NA	3.2 (0)	0.6 (0.7)	NA	0.1 (0)	0 (0)	NA
Snacks	13.9 (8.4)	13 (6)	0.25	2.3 (2.3)	1.5 (1)	0.01	0 (0.1)	0 (0)	< .001
Syrups/Spreads	0 (0)	0 (0)	0.30	0 (0)	0 (0)	0.42	0 (0)	0 (0)	0.30
Toaster Pastries	7.1 (1.6)	NA	NA	2.5 (0.9)	NA	NA	0.1 (0.1)	NA	NA
**OVERALL**	**4 (7.9)**	**7.7 (10)**	**< .001**	**1.2 (3.2)**	**2.4 (5.4)**	**< .001**	**0 (0.1)**	**0 (0.2)**	**< .001**
**Food Category**	Nutrient composition per TRA Reference Amount^2^								
	**Total Sugars (g)**			**Free Sugars (g)**			**Protein (g)**		
	**Median (IQR)**		**p value** ^**3**^	**Median (IQR)**		**p value** ^**3**^	**Median (IQR)**		**p value** ^**3**^
	**Child-appealing**	**Non-child-appealing**		**Child-appealing**	**Non-child-appealing**		**Child-appealing**	**Non-child-appealing**	
Cakes	36.9 (3)	25.1 (12.7)	< .001	34 (2.8)	22.9 (11.8)	0.001	2.4 (0.9)	3 (1.3)	0.04
Candy	21 (6.1)	18.7 (5.9)	< .001	21 (6.5)	18.3 (6.9)	< .001	1 (2)	2.4 (1.6)	< .001
Cereal	9.3 (3.7)	5 (4.5)	< .001	8.1 (3.9)	3.5 (4.1)	< .001	2 (1.1)	2.1 (1.3)	0.003
Cheese	0 (0.5)	0 (0)	0.58	0 (0)	0 (0)	0.43	7.1 (4.6)	7 (2.2)	0.31
Cookies	9.1 (3.1)	10 (3.6)	0.12	9.1 (3.1)	10 (4)	0.26	1.6 (0.9)	1.8 (0.9)	0.12
Crackers	1.3 (2.5)	1.5 (1.5)	0.07	0 (2.3)	1.3 (1.7)	0.05	3 (0)	1.7 (0.6)	< .001
Drinkable Yogurt	18.2 (0)	19.6 (3.9)	NA	12.8 (0)	14.2 (4.3)	NA	4 (0)	6 (1.5)	NA
Fruit Sauce	14.7 (1)	10.9 (6)	0.19	4.8 (4.9)	0 (6)	0.09	0.4 (1)	0.2 (0.2)	0.02
Grain Bars	9.2 (6.6)	9.3 (5.1)	0.89	8.7 (7)	8.4 (5.2)	0.71	1.7 (1.2)	2.3 (1)	0.10
Hot Cocoa	27.1 (0)	27.1 (13)	NA	24.2 (0)	24.2 (13.2)	NA	2.9 (0)	1.4 (2.9)	NA
Ice Cream	18 (8.8)	21.1 (8.2)	0.01	12.7 (6.3)	14.9 (6.3)	0.01	1.5 (2.7)	3 (2.1)	< .001
Juice	25 (13.7)	25 (6.8)	0.37	25 (13.7)	25 (6.8)	0.37	0 (0.4)	0.2 (1)	0.01
Meals	10.2 (7.2)	5.9 (4.9)	< .001	3.4 (11.2)	2 (5.7)	0.05	16.5 (11.9)	16.8 (7.9)	0.57
Meats	2.4 (1.9)	0 (1.1)	NA	1 (1.3)	0 (0.7)	NA	6 (7.4)	13.3 (5.7)	NA
Milk	24.5 (7)	11 (6)	< .001	13.1 (7.3)	0 (6)	< .001	8 (3.9)	7 (8)	0.05
Nut Butter	1.2 (1.6)	1 (0.1)	0.002	0.5 (1.8)	0 (0.5)	0.002	3 (0.8)	3 (1.2)	0.25
Pudding	18.4 (7.7)	18 (6.5)	0.43	14.8 (7.4)	16 (6.8)	0.04	1.3 (0.8)	2.6 (2.4)	0.003
Shakes	32.8 (3.2)	16 (12.1)	NA	32.8 (3.2)	16 (12.1)	NA	8.5 (0.4)	10.8 (7.8)	NA
Snacks	1.1 (1)	1 (2.2)	0.67	0 (0.8)	0.5 (1.7)	0.08	3.7 (1)	3 (1)	0.34
Syrups/Spreads	16 (6)	16 (4.4)	0.01	16 (6)	16 (6)	0.01	0 (0.2)	0 (0.2)	0.81
Toaster Pastries	10.2 (8.4)	NA	NA	7.8 (8.9)	0 (0)	NA	2 (0.2)	NA	NA
**OVERALL**	**14.7 (13)**	**9 (16.9)**	**< .001**	**11.5 (13.5)**	**6.2 (16)**	**< .001**	**2 (2.2)**	**3.1 (6.3)**	**< .001**
**Food Category**	Nutrient composition per TRA Reference Amount^2^								
	**Fibre (g)**			**Iron (mg)**			**Calcium (mg)**		
	**Median (IQR)**		**p value** ^**3**^	**Median (IQR)**		**p value** ^**3**^	**Median (IQR)**		**p value** ^**3**^
	**Child-appealing**	**Non-child-appealing**		**Child-appealing**	**Non-child-appealing**		**Child-appealing**	**Non-child-appealing**	
Cakes	1.2 (1.4)	1.1 (1.1)	0.72	1 (0.4)	1.1 (0.9)	0.96	28.1 (59.4)	33.5 (22.2)	0.58
Candy	0 (0)	1.1 (1.3)	< .001	0 (0.3)	0.7 (1.1)	< .001	0 (0)	29.3 (58.7)	< .001
Cereal	2 (1.1)	2 (2)	0.33	3.9 (3.4)	3.9 (3.1)	1.00	0 (110)	18.9 (66)	0.82
Cheese	0 (0)	0 (0)	0.61	0 (0)	0 (0)	0.38	157.1 (47.1)	220 (55)	0.02
Cookies	1 (0.5)	1 (1.2)	0.68	0.8 (0.5)	0.7 (0.5)	0.08	0 (19.7)	0 (22)	0.70
Crackers	1.3 (1.5)	1.5 (2.1)	0.01	1.2 (0.5)	0.6 (0.3)	0.01	33 (8.9)	40.1 (43.5)	0.34
Drinkable Yogurt	0 (0)	0 (0)	NA	0 (0)	0 (0)	NA	177.9 (0)	206.8 (47.1)	NA
Fruit Sauce	1.2 (0.8)	1.8 (0.9)	0.83	0.3 (0.7)	0 (0)	< .001	0 (0)	0 (0)	0.71
Grain Bars	1.5 (1.9)	2.3 (1.7)	0.06	0.9 (0.6)	0.7 (0.4)	0.03	23.6 (33.8)	23.8 (21.2)	0.17
Hot Cocoa	1.4 (0)	1.4 (0.4)	NA	0.8 (0)	1.2 (1)	NA	94.3 (0)	62.9 (91.2)	NA
Ice Cream	0 (1.5)	0 (1.5)	0.89	0.4 (0.8)	0.4 (1)	0.18	33.1 (66.2)	66.2 (60.0)	< .001
Juice	0 (0)	0 (0)	< .001	0 (0)	0 (0.3)	< .001	0 (0)	22 (22)	< .001
Meals	2.6 (2.2)	3.3 (2.5)	0.03	2.8 (1.2)	2.5 (1.9)	0.74	125.8 (182)	119.5 (161.1)	0.80
Meats	1 (0.8)	0.7 (1.6)	NA	1.1 (0.4)	1.4 (0.9)	NA	44 (11.3)	20.8 (22.3)	NA
Milk	0 (0)	0 (1)	0.17	0.6 (1.1)	0.3 (0.8)	0.37	302.5 (55)	330 (0)	0.05
Nut Butter	1 (0.1)	1 (0.1)	0.69	0.3 (0.2)	0.3 (0.2)	0.44	0 (10.3)	0 (20.6)	0.64
Pudding	1.3 (1.3)	0 (0.4)	0.01	0 (1.1)	0 (0.3)	0.14	28.9 (0)	57.8 (94.1)	0.38
Shakes	0.8 (0.7)	2.6 (2.1)	NA	0.3 (0.2)	0.7 (1.7)	NA	291.3 (22.2)	289.5 (66.1)	NA
Snacks	2 (2.4)	2 (1.8)	0.02	0.8 (0.6)	0.8 (0.5)	0.75	22 (44)	22 (44)	0.46
Syrups/Spreads	0 (0)	0 (0)	0.48	0 (2.1)	0 (0)	0.01	0 (0)	0 (0)	0.20
Toaster Pastries	1 (0.1)	NA	NA	1.1 (0.2)	NA	NA	0 (0)	NA	NA
**OVERALL**	**0.9 (1.5)**	**1 (2.3)**	**< .001**	**0.6 (1.2)**	**0.6 (1.4)**	**< .001**	**14.7 (51)**	**40.4 (105.7)**	**< .001**

^1^There was missing data for n = 12 products for saturated fat, n = 21 for trans fat, n = 1 for total sugars, n = 167 for free sugars, n = 9 for fibre, n = 4 for calcium and n = 5 for iron

^2^Reference amounts derived from Health Canada’s Table of Reference Amounts for Food, available from: https://www.canada.ca/en/health-canada/services/technical-documents-labelling-requirements/table-reference-amounts-food/nutrition-labelling.html

^3^Mann Whitney U tests compared the distribution of nutrients between products with child-appealing and non-child-appealing packaging where sample sizes were ≥10, p < .05 was considered significant.

In terms of ‘positive’ nutrients, products with child-appealing packaging were lower in protein (2± 2.2 g/RA vs. 3.1± 6.3 g/RA; p < .001), fibre (0.9± 1.5 mg/RA vs. 1± 2.3 mg/RA; p<0.001), iron (0.6± 1.2 mg/RA vs. 0.6± 1.4 mg/RA; p < .001), and calcium (14.7± 51 mg/RA vs. 40.4± 105.7 mg/RA; p < .001) compared to products with non-child-appealing packaging. Nutrient composition per RA between products with child-appealing and non-child-appealing packaging varied greatly by nutrient and by food category, and full results are presented in Table 5.

### Correlation between nutrient composition and marketing power score

Overall, there was a weak correlation between MPS and all nutrients that were assessed: energy per RA (*r*_*s*_ -0.00) sodium per RA (*r*_*s*_ -0.03), total fats per RA (*r*_*s*_ -0.05), saturated fats per RA (*r*_*s*_ -0.06), trans fats per RA (*r*_*s*_ -0.13), total sugars per RA (*r*_*s*_. 0.03), free sugars per RA (*r*_*s*_ -0.02), protein per RA (*r*_*s*_ -.0.07), fibre per RA (*r*_*s*_ 0.08), iron per RA (*r*_*s*_ 0.05) and calcium per RA (*r*_*s*_ -0.03) per RA (**Table 6**). The relationship between MPS and nutrient composition varied per food category and per nutrient, and full results are presented in Table 6. For example, in Cereals, there was a moderate positive correlation between both total sugars (*r*_*s*_ 0.54) and free sugars (*r*_*s*_ 0.52), and MPS and moderate negative correlations were seen between calcium and MPS (*r*_*s*_ -0.37) in Drinkable Yogurt and between trans fats and MPS (*r*_*s*_ -0.34) in Cakes. However, in most cases, there was weak correlation between MPS and any nutrient.

**Table 6:** Spearman correlation (*r*_*s*_)^1^ between nutrient composition and marketing power score^2^, overall and per food category.

**Food Category**	Total^3^	**Child-appealing packaging**	**Non-child-appealing packaging**		**Energy (kcal)**				**Sodium (mg)**	
	**n**	**n**	**n**	***r***_***s***_	**p value**		***r***_***s***_		**n**	
Cakes	155	10	145	0.03	0.701		0.04		0.631	
Candy	573	151	422	-0.22	< .001		0.09		0.027	
Cereal	88	47	41	0.12	0.250		-0.12		0.257	
Cheese	569	11	558	-0.07	0.110		0.15		< .001	
Cookies	523	84	439	-0.17	< .001		0.02		0.571	
Crackers	66	28	38	0.14	0.270		0.00		0.996	
Drinkable Yogurt	60	6	54	0.31	0.015		-0.18		0.160	
Fruit Sauce	65	17	48	-0.10	0.433		-0.21		0.090	
Grain Bars	199	30	169	0.05	0.441		0.03		0.654	
Hot Cocoa	32	1	31	0.10	0.601		-0.44		0.013	
Ice Cream	499	99	400	-0.17	< .001		-0.06		0.171	
Juice	608	56	552	-0.10	0.017		-0.03		0.526	
Meals	1012	66	946	0.03	0.317		0.03		0.292	
Meats	214	7	207	-0.08	0.229		0.04		0.558	
Milk	201	18	183	0.09	0.210		-0.05		0.483	
Nut Butter	101	23	78	-0.26	0.008		0.21		0.036	
Pudding	180	17	163	0.21	0.004		0.18		0.017	
Shakes	25	7	18	0.33	0.112		-0.25		0.224	
Snacks	577	38	539	0.09	0.028		0.00		0.964	
Syrups/Spreads	92	19	73	0.07	0.515		-0.01		0.907	
Toaster Pastries	11	11	0	-0.28	0.411		0.00		1.000	
**TOTAL**	**5850**	**746**	**5104**	**0.00**	**0.802**		**-0.03**		**0.014**	
**Food Category**	Total Fats (g)		Saturated Fats (g)		Trans Fats (g)		Total Sugars (g)		Free Sugars (g)	
	***r***_***s***_	**p value**	***r***_***s***_	**p value**	***r***_***s***_	**p value**	***r***_***s***_	**p value**	***r***_***s***_	**p value**
**Cakes**	-0.07	0.404	-0.13	0.111	-0.34	< .001	0.03	0.671	0.12	0.239
**Candy**	-0.22	< .001	-0.20	< .001	-0.15	< .001	0.03	0.489	0.01	0.857
**Cereal**	0.38	< .001	0.29	0.007	0.06	0.55	0.54	< .001	0.52	< .001
**Cheese**	-0.15	< .001	-0.15	< .001	-0.05	0.27	-0.16	< .001	-0.09	0.026
**Cookies**	-0.23	< .001	-0.16	< .001	-0.16	< .001	0.09	0.051	0.16	< .001
**Crackers**	-0.01	0.961	0.00	0.973	-0.16	0.20	-0.53	< .001	-0.52	< .001
**Drinkable Yogurt**	0.31	0.015	0.23	0.077	-0.21	0.11	0.27	0.036	0.21	0.111
**Fruit Sauce**	0.51	< .001	0.47	< .001	NA	NA	-0.18	0.160	-0.16	0.203
**Grain Bars**	0.01	0.926	-0.01	0.890	0.02	0.81	-0.05	0.465	-0.01	0.926
**Hot Cocoa**	0.08	0.668	-0.08	0.657	NA	NA	-0.09	0.612	-0.10	0.580
**Ice Cream**	-0.19	< .001	-0.17	< .001	-0.22	< .001	-0.15	0.001	-0.14	0.002
**Juice**	0.07	0.086	0.07	0.071	NA	NA	-0.08	0.047	-0.08	0.042
**Meals**	0.00	0.999	0.09	0.006	0.02	0.63	0.11	0.001	0.09	0.005
**Meats**	-0.08	0.216	-0.05	0.500	-0.09	0.19	-0.08	0.264	-0.02	0.809
**Milk**	-0.05	0.474	0.07	0.353	0.30	< .001	0.05	0.448	-0.12	0.080
**Nut Butter**	-0.28	0.005	-0.04	0.676	NA	NA	0.24	0.015	0.30	0.003
**Pudding**	0.34	< .001	0.32	< .001	0.10	0.20	-0.02	0.767	-0.11	0.138
**Shakes**	0.44	0.028	0.38	0.063	0.32	0.12	0.37	0.066	0.37	0.066
**Snacks**	0.07	0.107	-0.05	0.209	-0.16	< .001	-0.04	0.286	-0.02	0.591
**Syrups/Spreads**	-0.04	0.733	-0.03	0.808	-0.06	0.54	0.08	0.450	0.10	0.365
**Toaster Pastries**	0.22	0.525	0.22	0.514	0.29	0.39	-0.05	0.880	-0.05	0.880
**TOTAL**	**-0.05**	**< .001**	**-0.06**	**< .001**	**-0.13**	**< .001**	**0.03**	**0.011**	**0.02**	**0.065**
**Food Category**	Protein (g)		Fibre (g)		Iron (mg)			Calcium (mg)		
	***r***_***s***_	**p value**	***r***_***s***_	**p value**	***r***_***s***_		**p value**		***r***_***s***_	**p value**
**Cakes**	0.05	0.511	0.18	0.028		0.05		0.519	-0.04	0.628
**Candy**	-0.15	< .001	-0.14	0.001		-0.11		0.006	-0.19	< .001
**Cereal**	-0.12	0.266	0.15	0.162		-0.17		0.123	0.24	0.022
**Cheese**	0.15	< .001	0.07	0.109		-0.03		0.488	0.19	< .001
**Cookies**	-0.12	0.005	0.11	0.010		0.14		0.001	-0.03	0.462
**Crackers**	0.26	0.032	-0.17	0.178		0.27		0.026	-0.16	0.211
**Drinkable Yogurt**	-0.30	0.018	-0.02	0.903		-0.25		0.052	-0.37	0.004
**Fruit Sauce**	0.51	< .001	-0.17	0.170		0.44		< .001	-0.04	0.769
**Grain Bars**	0.13	0.073	-0.13	0.074		0.08		0.252	-0.10	0.168
**Hot Cocoa**	0.52	0.002	0.28	0.118		0.23		0.206	0.52	0.002
**Ice Cream**	-0.19	< .001	-0.02	0.734		-0.02		0.661	-0.30	< .001
**Juice**	0.05	0.210	0.07	0.071		-0.03		0.473	-0.01	0.865
**Meals**	-0.02	0.533	0.07	0.037		0.00		0.895	0.14	< .001
**Meats**	-0.07	0.285	0.07	0.317		-0.09		0.183	0.12	0.082
**Milk**	0.17	0.014	-0.32	< .001		-0.16		0.023	0.02	0.728
**Nut Butter**	-0.149	0.137	-0.08	0.412		0.02		0.869	0.18	0.069
**Pudding**	0.23	0.002	0.12	0.101		0.11		0.138	0.32	< .001
**Shakes**	-0.31	0.136	-0.42	0.039		0.07		0.740	-0.27	0.188
**Snacks**	-0.04	0.323	0.09	0.033		-0.09		0.028	0.00	0.949
**Syrups/Spreads**	0.16	0.136	0.02	0.854		0.32		0.002	0.19	0.067
**Toaster Pastries**	-0.28	0.411	-0.29	0.389		-0.28		0.411	-0.15	0.662
**TOTAL**	**-0.07**	< .001	**0.08**	< .001		**0.05**		< .001	**-0.03**	**0.010**

^1^Values of Spearman’s *r*_*s*_ were interpreted as follows: 0.00 as “zero”, 0.01–0.30 as “weak”, 0.31–0.60 as “moderate”, 0.61–0.99 as “strong”, and 1.00 as “perfect”; ^2^Marketing power score = the total number of core and broad techniques displayed on the package; ^3^There was missing data for n = 12 products for saturated fat, n = 21 for trans fat, n = 1 for total sugars, n = 167 for free sugars, n = 9 for fibre, n = 4 for calcium and n = 5 for iron.

## Discussion

The aim of this study was to evaluate the presence, type, and power of child-appealing marketing on product packaging, as well as compare the nutritional quality of products with child-appealing versus non-child-appealing packaging and determine the relationship between nutritional quality and marketing power. The results of this work showed that child-appealing marketing is prevalent on product packaging, especially in certain food categories (either by proportion or by absolute number of products), such as Toaster Pastries, Cereal, Crackers, Candy, Cookies, Ice Cream, Meals, and Juice.

The specific marketing techniques that were displayed on product packages varied across food categories; however, core techniques that have traditionally been found to be used in child-appealing marketing, such as having a child-appealing visual design, appeals to fun or cool and the use of characters remained popular across the sample in the present study [25, 27, 30]. Another important finding was that broad techniques (i.e., techniques that don’t make a product child-appealing on their own but increase marketing power) were displayed much more frequently than core techniques, with over 90% of products displaying at least one broad technique (versus only approximately 13% displaying core techniques). This is important given that these are techniques that would not be captured under marketing restrictions as they are not specifically ‘directed at children’, but still increase the product’s appeal to children [26]. Previous work has shown that broad techniques such as appeals to health, nutrition, and taste are attractive to children and this study found that these were the most frequently displayed techniques overall [26]. These results also corroborate evidence speaking to the highly marketed nature of the Canadian food supply more generally (i.e., not just child-appealing marketing), which is concerning given that there are currently no regulations on the healthfulness of products that are allowed to display nutrition marketing or other marketing, as is mandated by certain countries (e.g., Australia and New Zealand) [45, 46].

While the power of marketing (i.e., the number of techniques displayed) also varied between food categories, the food categories that were most powerfully marketed on average–Cereal, Crackers, Toaster Pastries, etc.–were also among those most frequently displaying child-appealing marketing. These results indicate that in packaged foods, there are certain food categories that are particularly problematic with regards to both the power and children’s potential exposure to child-appealing marketing, both of which are metrics the WHO considers critical in monitoring and regulating food marketing [6, 8, 16].

This study also showed that based on the nutrient profile model Health Canada has proposed to underpin marketing restrictions in Canada, almost all products would be restricted from being advertised to children, but significantly more products with child-appealing packaging would be restricted compared to non-child-appealing packaging (98% vs 94%). These results reinforce the necessity of implementing restrictions on the marketing of unhealthy foods to children in Canada and also ensuring that marketing on product packaging is included within the scope of the regulations, which was not the case in the original Health Canada proposal [42]. Importantly as well, the subset of products that were found to both have child-appealing packaging and be restricted from marketing were also the most powerfully marketed group of products on average (i.e., vs. products with child-appealing packaging that would be permitted or products non-child-appealing packaging that would be either restricted or permitted). This is positive given that this is the subset of products that would be directly impacted by Health Canada’s proposed regulations, meaning that their policy would result in the restriction of child-appealing advertising of the most powerfully marketed unhealthy products in the packaged food supply–again, with the important caveat that packaging be included within the scope.

This study found no major overall differences in nutrient composition between products with child-appealing and non-child-appealing packaging and weak correlations between nutrient levels and marketing power, indicating that when looking at packaged foods altogether, the presence and power of child-appealing marketing was not necessarily indicative of nutritional composition. However, there were some trends in key food categories and nutrients that merit discussion. For example, in the Cereals category, there was no difference in energy, sodium or saturated fats between products with child-appealing and non-child-appealing packaging; however, cereals with child-appealing packaging had significantly higher levels of total sugars and free sugars, and lower levels of protein than cereals with non-child-appealing packaging. Cereals that were more powerfully marketed were also more likely to be higher in total and free sugars.

Total sugars were a nutrient of particular concern across the board, with 9/21 food categories showing significantly higher total sugars in products with child-appealing packaging, and significantly more products with child-appealing packaging exceeding the Health Canada sugars threshold compared to non-child-appealing packaging (80% vs. 54%). A similar trend was seen with regards to free sugars, aligning with previous analyses that have shown that child-appealing marketing is more likely to occur on products with excess levels of free sugars [47]. These findings reiterate the importance of considering free sugars alongside total sugars within Canadian nutrient policies and regulations [47]. Moreover, it is clear that food manufacturers are marketing sugary foods to children, which is highly problematic given that child-appealing marketing shapes children’s taste preferences, potentially predisposing them to an affinity for sweetness early in life and putting them at risk for excess consumption of sugars over the life course and thus increasing their risk for dental caries, obesity and associated chronic disease [1, 3, 4, 8].

While more products with non-child-appealing packaging exceeded Health Canada’s sodium threshold than child-appealing packaging (54% vs. 42%), when looking directly at nutrient composition, in every food category, products with child-appealing packaging either had higher than or similar amounts of sodium than products with non-child-appealing packaging, indicating that sodium is also an important nutrient to continue monitoring in this context and in the food supply overall.

There were some food categories included in the sample that are considered as “core” components of a diet (rather than “junk” type foods and snacks) such as milks, meats, and meals, that should ideally be providing important nutrients to the diet, in particular those products being aimed at children. This study found that this was not always the case. For instance, child-appealing milk products were higher in energy, saturated fats, and total and free sugars than non-child-appealing milks and tended to be sweetened and flavoured varieties. While they were higher in protein, they were significantly lower in calcium on average–a nutrient that is integral to children’s growth and development. Similarly, child-appealing meat products were lower in protein than non-child-appealing meats, while being higher in sodium and total/free sugars. Child-appealing pre-prepared meal products followed the same trend, being higher in most ‘negative’ nutrients (i.e., energy, sodium, sat fat, total/free sugars) while providing no difference in ‘positive’ nutrients (protein, calcium, iron) and even being lower in fibre than non-child-appealing meals. Therefore, it seems that child-appealing core foods are not only contributing a significant amount of ‘negative’ nutrients but are also lower in the ‘positive’ nutrients that should be emphasized in children’s diets. In sum, the results of this work contribute to the growing international body of literature speaking to the poor nutritional quality of food and beverage products that are being marketed to children, regardless of the marketing platform (e.g., on television, digital or other media) or how product healthfulness is evaluated (e.g., with various nutrient profile models) [4, 5, 7].

This study presents an exciting step forward in terms of child-appealing food and beverage marketing assessment methodologies as it utilizes the newly developed and validated CAP tool. As such, this is the first study (to our knowledge) to empirically measure the power of food marketing. The use of the CAP tool facilitated the documentation of how child-appealing marketing on packaging employs a much broader range of marketing techniques than has previously been tracked. These data will provide a critical addition to efforts aimed at monitoring how marketing practices evolve over time and/or in response to restrictions [8]. There are, however, some limitations inherent to the application of the CAP tool [26]. Despite high-interrater reliability between coders during internal validity testing, there is a certain degree of subjectivity involved in the process of coding of marketing techniques that can result in minor inconsistencies. Additionally, the CAP tool does not account for the fact that certain marketing techniques may be more persuasive than others or that certain product types may be appealing to children regardless of the marketing displayed on the package (e.g., cookies, candy). Therefore, this study likely underestimates the proportion of the packaged food supply that appeals to children, as well as the strength of this appeal.

This was also the first study to examine the relationship between marketing power and product healthfulness. While this study did not discern a clear relationship between nutritional quality and MPS, in part due to the low variability in MPS and poor nutritional quality of the sample overall, MPS is still an important indicator of the persuasiveness of the marketing message that is being promoted. Moreover, if MPS is adapted and used in assessments of food marketing on platforms where there is potential for greater variability in marketing power (e.g., on digital media), a stronger relationship between nutritional quality and MPS could emerge. It is also possible that due to the overall poor nutritional quality and highly-processed nature of the Canadian packaged food supply, that major differences in nutritional quality/composition between products of different MPS were difficult to discern.

Overall, this study provides a comprehensive assessment and important baseline (i.e., pre-regulation) of the nutritional quality and composition of a large sample of child-appealing packaged products. Moreover, this study assessed products’ nutritional composition in terms of not only ‘negative’ nutrient, but also ‘positive’ nutrients which allowed for the examination of products’ with child-appealing packaging potentially beneficial contributions to the diet, which is not typically the focus of studies in this field. The FLIP database, however, is cross sectional, meaning that seasonal or limited-time marketing promotions occurring outside of when data collection occurred would not have been captured in this study. Additionally, FLIP is not sales-weighted, meaning that results can only describe the marketing and nutritional quality of products that are available to consumers and cannot be extrapolated to infer children’s purchasing or consumption patterns. Future studies using sales-weighted data will be integral to elucidating how products with child-appealing packaging (and the power of their marketing) directly impact children’s diet-quality.

## Conclusions

Overall, this study found that child-appealing marketing is pervasive on product packaging with varying degrees of marketing power and with variability in the specific techniques being used. Results highlighted that there are certain food categories that are particularly problematic in terms of both the prevalence and the power of child-appealing marketing, speaking to the importance of including product packaging within food marketing regulations. While this study found variability in nutritional quality and composition depending on the food category and the nutrient, results showed that in many cases, products with child-appealing packaging were higher in nutrients of concern–in particular, total sugars, free sugars, and sodium—than products with non-child-appealing packaging. Moreover, core foods with child-appealing packages do not appear to be good sources of nutrients to be encouraged within children’s diets. More products with child-appealing packaging than non-child-appealing packaging—especially those displaying more powerful marketing–exceeded Health Canada’s nutrient thresholds and would therefore be restricted from being advertised. If packaging is included within the scope, Health Canada’s proposed regulations could be effective in restricting children’s exposure to the most powerfully marketed and less healthy packaged foods and should absolutely be implemented.

## Supporting information

S1 File This file contains all supporting tables. (DOCX)
