# Supplementary material for: Child-appealing packaged food and beverage products in Canada–Prevalence, power, and nutritional quality
Source: PLoS One. 2023 May 3;18(5):e0284350. doi: 10.1371/journal.pone.0284350 (PMC10156002; doi:10.1371/journal.pone.0284350)
Supplement: S1 File — (DOCX) [file pone.0284350.s001.docx]

**Supplementary Table S1.** Description of food categories used for analysis

| Food Category Name | Number of products | TRA subcategories included^1^ | Description of included products^1^ |
| --- | --- | --- | --- |
| Cookies | 523 | A10 | Cookies, with or without coating or filling; graham wafers |
| Crackers | 66 | A12 | Snack crackers, crackers and cheese dip pack |
| Toaster Pastries | 11 | A14 | Toaster pastries |
| Grain bars | 199 | A18, A19 | Grain-based bars with or without filling or partial or full coating |
| Cakes | 155 | A6 | Medium weight cake, such as cake with or without icing or filling, cake with less than 35% of the finished weight as fruit, nuts or vegetables or any of these combined; light weight cake with icing; Boston cream pie, cupcakes, eclairs, or cream puffs, individually wrapped snack cakes such as Swiss rolls. |
| Hot Cocoa | 32 | B5 | Cocoa and chocolate beverages (hot) |
| Cereal | 88 | C3 | Ready-to-eat breakfast cereals, puffed and coated, flaked, extruded, without fruit or nuts (weighing 20 g to 42 g per 250 mL), very high fibre cereals (with 28 g or more fibre per 100 g) |
| Cheese | 569 | D1 | Cheese, including cream cheese and cheese spread |
| Milk | 201 | D11 | Milk, buttermilk and milk-based drinks, such as chocolate milk, plant-based beverages |
| Drinkable Yogurt | 60 | D12 | Fermented dairy drinks including drinkable yogurts, kefir |
| Shakes | 25 | D13 | Shakes, with or without coffee or juice, including protein shakes and dairy substitute shakes, smoothies (if whey/dairy or plant-based beverage is a main ingredient), milkshakes |
| Ice cream | 499 | E1, E2, E3, E4 | Ice cream, ice milk, frozen yogurt, sherbet, other frozen dairy and non-dairy desserts sold in tubs, cakes, sandwiches, cones, pops, bars or cups, including sorbet and gelato; Sundaes |
| Pudding | 180 | E5 | Custard, gelatin and pudding |
| Juice | 608 | J11 | Juices, nectars and fruit drinks represented for use as substitutes for fruit juices, juice-based smoothies |
| Fruit Sauce | 65 | J6 | Apple (or other fruit) sauces, including those that contain other fruit and vegetables |
| Meats | 214 | L7 | Patties, meatballs, sausage meat and ground meat, with or without breading or batter, corn dog on a stick (breaded), falafels, including simulated meat and poultry products |
| Meals | 1012 | N1, N2 | Combination dishes, such as macaroni and cheese, spaghetti with sauce, lasagna, stir fry, casserole, wieners and beans, chili, ravioli with sauce, beef stroganoff, poultry à la king, stew, poutine, butter chicken with rice, shepherd’s pie, burritos, pizza, pastry rolls, quiche, sandwiches, crackers and meat or poultry lunch-type packages, burger on a bun, frank on a bun, calzones, tacos, pockets stuffed with meat, empanadas, fajitas, sushi, souvlaki, pot pie |
| Nut Butter | 101 | O3 | Peanut butter, nut butters and substitutes, such as coconut and soya butter |
| Snacks | 577 | S1 | Chips, pretzels, popcorn, extruded snacks, grain and pulse-based snacks, pita chips and fruit-based snacks, such as fruit chips |
| Candy | 573 | U1, U11, U10 | Candies, confectionaries and chocolates, including a mixture of these with or without other food added, chocolate bars and other chocolate products, Halloween candy, box of assorted chocolates, Marshmallows; Fruit leather, bar or mini pieces, that may or may not contain vegetable ingredients |
| Syrups/Spreads | 92 | U8, U15 | Honey, molasses, and bread spreads; syrups used as ingredients, such as corn syrup, agave syrup and flavoured syrups for milk |

^1^Subcategories and descriptions adapted from Health Canada’s Table of Reference Amounts for Food, available from: <https://www.canada.ca/en/health-canada/services/technical-documents-labelling-requirements/table-reference-amounts-food/nutrition-labelling.html>

**Supplementary Table S2.** Proportion of products displaying each core and broad marketing technique^1^, overall and by food category

|  | Core marketing techniques | | | | | | | | | | | | % displaying any core technique |
| --- | --- | --- | --- | --- | --- | --- | --- | --- | --- | --- | --- | --- | --- |
| Food Category | **Visual/ graphical design** | **Unconventional shape** | **Unconventional flavour** | **Unconventional colour** | **Games or activities** | **Branded characters** | **Licensed Characters** | **Celebrities** | **Other tie-ins** | **Coupons, Contests, Prizes, Toys** | **Fun/Cool** | **Websites/**  **Social Media Etc.** |  |
| Cakes | 4.5% | 0.0% | 0.0% | 1.3% | 0.0% | 1.3% | 0.0% | 0.0% | 0.0% | 0.0% | 1.3% | 0.0% | 6.5% |
| Candy | 22.2% | 5.8% | 2.4% | 0.5% | 0.2% | 5.6% | 0.9% | 0.0% | 0.0% | 0.3% | 6.3% | 3.7% | 26.4% |
| Cereal | 44.3% | 10.2% | 4.5% | 9.1% | 11.4% | 15.9% | 4.5% | 0.0% | 0.0% | 1.1% | 19.3% | 6.8% | 53.4% |
| Cheese | 1.9% | 0.0% | 0.5% | 0.0% | 0.0% | 0.7% | 0.0% | 0.0% | 0.0% | 0.0% | 0.7% | 0.7% | 1.9% |
| Cookies | 13.2% | 9.0% | 1.1% | 0.2% | 1.0% | 2.5% | 0.8% | 0.0% | 0.0% | 0.4% | 5.0% | 2.9% | 16.1% |
| Crackers | 40.9% | 30.3% | 12.1% | 1.5% | 9.1% | 25.8% | 1.5% | 0.0% | 0.0% | 0.0% | 10.6% | 13.6% | 42.4% |
| Drinkable Yogurt | 8.3% | 0.0% | 0.0% | 0.0% | 0.0% | 1.7% | 1.7% | 0.0% | 0.0% | 3.3% | 1.7% | 3.3% | 10.0% |
| Fruit Sauce | 26.2% | 0.0% | 4.6% | 0.0% | 0.0% | 0.0% | 0.0% | 0.0% | 0.0% | 0.0% | 13.8% | 0.0% | 26.2% |
| Grain Bars | 12.1% | 0.5% | 0.5% | 0.5% | 2.5% | 2.5% | 4.5% | 0.0% | 3.5% | 3.5% | 5.0% | 2.5% | 15.1% |
| Hot Cocoa | 3.1% | 0.0% | 0.0% | 0.0% | 0.0% | 0.0% | 0.0% | 0.0% | 0.0% | 0.0% | 0.0% | 0.0% | 3.1% |
| Ice Cream | 15.1% | 1.1% | 3.6% | 1.9% | 0.0% | 4.2% | 0.3% | 0.2% | 0.2% | 0.0% | 3.8% | 5.9% | 19.8% |
| Juice | 8.6% | 0.0% | 2.6% | 0.0% | 0.0% | 0.0% | 0.7% | 0.0% | 0.0% | 1.0% | 2.8% | 1.2% | 9.2% |
| Meals | 3.2% | 1.5% | 0.0% | 0.0% | 0.0% | 2.6% | 1.5% | 0.0% | 0.1% | 0.0% | 2.4% | 1.1% | 6.5% |
| Meats | 2.8% | 0.9% | 0.0% | 0.0% | 0.0% | 0.0% | 0.5% | 0.0% | 0.0% | 0.0% | 1.4% | 0.0% | 3.3% |
| Milk | 5.5% | 0.0% | 2.0% | 0.0% | 0.0% | 1.5% | 0.0% | 0.0% | 0.0% | 0.0% | 1.0% | 0.0% | 9.0% |
| Nut Butter | 9.9% | 0.0% | 5.9% | 0.0% | 0.0% | 12.9% | 0.0% | 0.0% | 0.0% | 0.0% | 0.0% | 0.0% | 22.8% |
| Pudding | 9.4% | 0.0% | 0.0% | 0.0% | 0.0% | 0.0% | 0.0% | 0.0% | 0.0% | 0.0% | 0.0% | 0.0% | 9.4% |
| Shakes | 28.0% | 0.0% | 0.0% | 0.0% | 0.0% | 4.0% | 0.0% | 0.0% | 0.0% | 0.0% | 12.0% | 0.0% | 28.0% |
| Snacks | 4.7% | 0.2% | 0.2% | 0.0% | 0.2% | 4.2% | 0.0% | 0.0% | 0.0% | 0.2% | 1.6% | 0.0% | 6.6% |
| Syrups/Spreads | 16.3% | 0.0% | 0.0% | 0.0% | 0.0% | 8.7% | 1.1% | 0.0% | 0.0% | 0.0% | 5.4% | 0.0% | 20.6% |
| Toaster Pastries | 100.0% | 0.0% | 9.1% | 0.0% | 0.0% | 54.5% | 0.0% | 0.0% | 0.0% | 0.0% | 9.1% | 0.0% | 100.0% |
| OVERALL | **10.0%** | **2.3%** | **1.5%** | **0.4%** | **0.5%** | **3.2%** | **0.8%** | **0.0%** | **0.2%** | **0.4%** | **3.3%** | **1.9%** | **12.8%** |

**Supplementary Table S2.** Proportion of products displaying each core and broad marketing technique^1^, overall and by food category (cont’d)

|  | Broad marketing techniques | | | | | | | | | | % displaying any broad technique |
| --- | --- | --- | --- | --- | --- | --- | --- | --- | --- | --- | --- |
| Food Category | **Font/Lettering** | **Product Name** | **Logo or Images** | **Convenient packaging** | **Taste & Texture** | **Health & Nutrition** | **Product Benefits** | **Recipes** | **Websites /Social Media Etc.** | **Coupons, Contests, Giveaways** |  |
| Cakes | 11.6% | 3.9% | 14.2% | 1.3% | 36.8% | 27.1% | 11.0% | 0.0% | 15.5% | 0.0% | 56.8% |
| Candy | 3.7% | 3.1% | 2.4% | 1.0% | 47.3% | 58.6% | 23.7% | 1.0% | 27.7% | 0.2% | 85.3% |
| Cereal | 8.0% | 31.8% | 5.7% | 2.3% | 46.6% | 98.9% | 54.5% | 12.5% | 56.8% | 2.3% | 100.0% |
| Cheese | 0.9% | 2.8% | 4.0% | 1.9% | 22.0% | 62.4% | 29.7% | 8.6% | 19.9% | 1.2% | 86.3% |
| Cookies | 13.2% | 17.0% | 10.1% | 5.0% | 49.1% | 70.9% | 16.8% | 2.5% | 31.4% | 1.1% | 90.1% |
| Crackers | 13.6% | 37.9% | 1.5% | 25.8% | 54.5% | 80.3% | 6.1% | 0.0% | 10.6% | 0.0% | 97.0% |
| Drinkable Yogurt | 15.0% | 16.7% | 10.0% | 6.7% | 13.3% | 98.3% | 48.3% | 0.0% | 43.3% | 11.7% | 100.0% |
| Fruit Sauce | 18.5% | 33.8% | 0.0% | 27.7% | 30.8% | 90.8% | 16.9% | 0.0% | 18.5% | 0.0% | 92.3% |
| Grain Bars | 9.5% | 11.6% | 15.1% | 12.6% | 41.7% | 97.0% | 14.1% | 0.0% | 49.2% | 0.0% | 99.5% |
| Hot Cocoa | 0.0% | 0.0% | 9.4% | 0.0% | 81.3% | 71.9% | 31.3% | 3.1% | 37.5% | 0.0% | 96.9% |
| Ice Cream | 9.1% | 9.9% | 19.3% | 0.2% | 46.4% | 76.1% | 66.9% | 0.0% | 30.9% | 0.0% | 100.0% |
| Juice | 4.6% | 5.6% | 4.3% | 1.6% | 27.1% | 89.0% | 7.9% | 0.3% | 24.8% | 1.8% | 94.9% |
| Meals | 1.7% | 2.6% | 3.7% | 1.1% | 51.6% | 56.6% | 33.4% | 2.9% | 29.2% | 0.5% | 87.7% |
| Meats | 0.0% | 0.0% | 0.9% | 0.0% | 50.9% | 77.1% | 27.6% | 0.5% | 22.4% | 0.9% | 95.3% |
| Milk | 1.0% | 2.0% | 26.4% | 2.0% | 66.2% | 97.0% | 42.8% | 3.5% | 43.3% | 1.5% | 99.5% |
| Nut Butter | 2.0% | 3.0% | 11.9% | 7.9% | 27.7% | 90.1% | 5.9% | 9.9% | 30.7% | 0.0% | 97.0% |
| Pudding | 4.4% | 3.9% | 4.4% | 9.4% | 20.0% | 68.9% | 18.9% | 7.2% | 16.7% | 0.0% | 87.2% |
| Shakes | 12.0% | 12.0% | 12.0% | 8.0% | 20.0% | 100.0% | 12.0% | 0.0% | 12.0% | 0.0% | 100.0% |
| Snacks | 5.9% | 2.3% | 11.8% | 2.3% | 71.6% | 76.4% | 15.3% | 1.6% | 52.2% | 0.9% | 93.6% |
| Syrups/Spreads | 3.3% | 5.4% | 4.3% | 0.0% | 33.7% | 53.3% | 23.9% | 2.2% | 22.8% | 0.0% | 80.4% |
| Toaster Pastries | 0.0% | 36.4% | 0.0% | 0.0% | 9.1% | 90.9% | 0.0% | 0.0% | 0.0% | 0.0% | 100.0% |
| OVERALL | **5.3%** | **6.5%** | **8.1%** | **3.0%** | **44.5%** | **71.2%** | **26.7%** | **2.6%** | **30.3%** | **0.8%** | **90.8%** |

^1^Core and broad marketing techniques, as defined by the child-appealing packaging (CAP) coding tool (Appendix A)

**Supplementary Table S3.** Comparison of Marketing Power Scores (MPS)^1^ between products that would be permitted or restricted from advertising to children according to Health Canada's proposed nutrient thresholds for advertising restrictions^2^

|  | Permitted for advertising to children | | Restricted from advertising to children | | t-test statistic  (p-value) |
| --- | --- | --- | --- | --- | --- |
|  | **n** | **Mean (SD) MPS** | **n** | **Mean (SD) MPS** |  |
| Child-appealing products | 16 | 3.6 (1.1) | 730 | 4.2 (1.9) | t = 2.2 (p=0.04) |
| Non-child-appealing products | 295 | 2.3 (1.2) | 4809 | 1.9 (1.2) | t = 4.9 (p <.001) |
| OVERALL | **311** | **2.3 (1.2)** | **5539** | **2.2 (1.5)** | **t=1.6 (p=0.12)** |

^1^Marketing power score = the total number of core and broad techniques displayed on the package; ^2^Mulligan et al. (2020). Evaluating the Canadian packaged food supply using Health Canada’s proposed nutrient criteria for restricting food and beverage marketing to children. *IJERPH*, 17(4), 1250

**Supplementary Table S4.** Number and proportion of products that would exceed each of Health Canada's proposed nutrient thresholds for advertising restrictions^1^ in child-appealing and non-child-appealing products

| Food Category | Child-appealing products | | | | | | Non-child-appealing products | | | | | |
| --- | --- | --- | --- | --- | --- | --- | --- | --- | --- | --- | --- | --- |
|  | **Sodium threshold** | | **Total sugars threshold** | | **Saturated Fats threshold** | | **Sodium threshold** | | **Total sugars threshold** | | **Saturated Fats threshold** | |
|  | **n** | **%** | **n** | **%** | **n** | **%** | **n** | **%** | **n** | **%** | **n** | **%** |
| Cakes | 9 | 90.0% | 10 | 100.0% | 9 | 90.0% | 116 | 80.0% | 145 | 100.0% | 138 | 95.2% |
| Candy | 5 | 3.4% | 151 | 100.0% | 31 | 20.9% | 12 | 3.0% | 393 | 93.1% | 340 | 84.0% |
| Cereal | 39 | 83.0% | 42 | 89.4% | 0 | 0.0% | 35 | 85.4% | 27 | 65.9% | 0 | 0.0% |
| Cheese | 11 | 100.0% | 0 | 0.0% | 0 | 0.0% | 551 | 98.7% | 19 | 3.4% | 29 | 5.2% |
| Cookies | 47 | 56.0% | 82 | 97.6% | 39 | 46.4% | 217 | 49.4% | 428 | 97.5% | 301 | 68.6% |
| Crackers | 27 | 96.4% | 7 | 25.0% | 5 | 17.9% | 38 | 100.0% | 6 | 15.8% | 3 | 7.9% |
| Drinkable Yogurt | 0 | 0.0% | 6 | 100.0% | 0 | 0.0% | 1 | 1.9% | 45 | 83.3% | 0 | 0.0% |
| Fruit Sauce | 0 | 0.0% | 17 | 100.0% | 0 | 0.0% | 0 | 0.0% | 48 | 100.0% | 0 | 0.0% |
| Grain Bars | 6 | 20.0% | 28 | 93.3% | 5 | 16.7% | 36 | 21.3% | 164 | 97.0% | 58 | 34.3% |
| Hot Cocoa | 0 | 0.0% | 1 | 100.0% | 1 | 100.0% | 18 | 58.1% | 31 | 100.0% | 23 | 74.2% |
| Ice Cream | 10 | 10.1% | 99 | 100.0% | 53 | 53.5% | 34 | 8.5% | 396 | 99.0% | 225 | 56.3% |
| Juice | 2 | 3.6% | 56 | 100.0% | 0 | 0.0% | 1 | 0.2% | 539 | 97.6% | 0 | 0.0% |
| Meals | 66 | 100.0% | 19 | 28.8% | 26 | 39.4% | 914 | 96.6% | 119 | 12.6% | 381 | 40.3% |
| Meats | 7 | 100.0% | 0 | 0.0% | 1 | 14.3% | 184 | 88.9% | 1 | 0.5% | 15 | 7.2% |
| Milk | 11 | 61.1% | 16 | 88.9% | 1 | 5.6% | 41 | 22.4% | 76 | 41.5% | 4 | 2.2% |
| Nut Butter | 14 | 60.9% | 8 | 34.8% | 5 | 21.7% | 21 | 26.9% | 17 | 21.8% | 7 | 9.0% |
| Pudding | 12 | 70.6% | 13 | 76.5% | 1 | 5.9% | 61 | 37.4% | 143 | 87.7% | 15 | 9.2% |
| Shakes | 7 | 100.0% | 7 | 100.0% | 0 | 0.0% | 10 | 55.6% | 14 | 77.8% | 0 | 0.0% |
| Snacks | 31 | 81.6% | 2 | 5.3% | 19 | 50.0% | 463 | 85.9% | 55 | 10.2% | 109 | 20.2% |
| Syrups/Spreads | 0 | 0.0% | 19 | 100.0% | 1 | 4.5% | 1 | 1.1% | 71 | 96.7% | 7 | 7.8% |
| Toaster Pastries | 11 | 100.0% | 11 | 100.0% | 6 | 54.5% | NA | NA | NA | NA | NA | NA |
| OVERALL | **315** | **42.2%** | **594** | **79.6%** | **203** | **27.2%** | **2754** | **54.0%** | **2737** | **53.6%** | **1655** | **32.4%** |

^1^Mulligan et al. (2020). Evaluating the Canadian packaged food supply using Health Canada’s proposed nutrient criteria for restricting food and beverage marketing to children. *IJERPH*, 17(4), 1250

**Supplementary Table S5.** Mean Marketing Power Score (MPS)^1^ by the number of Health Canada's proposed nutrient thresholds^2^ exceeded

| Food Category | No nutrient thresholds exceeded | | 1 nutrient threshold exceeded | | 2 nutrient thresholds exceeded | | 3 nutrient thresholds exceeded | |
| --- | --- | --- | --- | --- | --- | --- | --- | --- |
|  | **n** | **Mean (SD) MPS** | **n** | **Mean (SD) MPS** | **n** | **Mean (SD) MPS** | **n** | **Mean (SD) MPS** |
| Cakes | 0 | NA | 2 | 1 (0) | 34 | 1.6 (1.5) | 119 | 1.2 (1.3) |
| Candy | 4 | 2.3 (1.3) | 217 | 2.5 (1.6) | 341 | 2 (1.4) | 11 | 2.1 (1.1) |
| Cereal | 2 | 1 (0) | 29 | 4.3 (1.9) | 57 | 4.7 (2) | 0 | NA |
| Cheese | 5 | 1.2 (1.1) | 518 | 1.6 (1.3) | 46 | 1.5 (1.1) | 0 | NA |
| Cookies | 5 | 3 (2.3) | 81 | 2.5 (1.8) | 278 | 2.7 (1.8) | 159 | 2.3 (1.7) |
| Crackers | 0 | NA | 51 | 4.1 (2.5) | 10 | 2.6 (2.4) | 5 | 2.4 (1.1) |
| Drinkable Yogurt | 8 | 2.3 (0.9) | 52 | 2.9 (1.5) | 0 | NA | 0 | NA |
| Fruit Sauce | 0 | NA | 65 | 2.8 (2.2) | 0 | NA | 0 | NA |
| Grain Bars | 6 | 2.7 (0.8) | 105 | 3 (2) | 72 | 2.8 (1.3) | 16 | 2.6 (1) |
| Hot Cocoa | 0 | NA | 5 | 4 (0) | 12 | 2.3 (0.8) | 15 | 1.9 (0.9) |
| Ice Cream | 3 | 3 (0) | 216 | 2.9 (1.2) | 239 | 2.9 (1.1) | 41 | 3 (1.2) |
| Juice | 13 | 1.5 (0.5) | 592 | 1.8 (1.1) | 3 | 2.7 (0.6) | 0 | NA |
| Meals | 21 | 2 (1.6) | 496 | 1.9 (1.3) | 456 | 1.8 (1.2) | 39 | 3.7 (2.6) |
| Meats | 23 | 1.3 (0.7) | 174 | 1.9 (0.9) | 17 | 2.2 (1) | 0 | NA |
| Milk | 93 | 3.2 (1) | 70 | 2.7 (0.8) | 35 | 3 (1.1) | 3 | 2 (0) |
| Nut Butter | 49 | 1.7 (0.7) | 32 | 2.5 (1.1) | 20 | 2.8 (2) | 0 | NA |
| Pudding | 15 | 1.6 (1.3) | 89 | 1.4 (1.1) | 72 | 1.8 (1.1) | 4 | 3.8 (0.5) |
| Shakes | 0 | NA | 12 | 2 (1.3) | 13 | 2.6 (1.7) | 0 | NA |
| Snacks | 62 | 2.6 (1.1) | 370 | 2.6 (1.1) | 126 | 2.4 (1.4) | 19 | 2.1 (1) |
| Syrups/Spreads | 2 | 0 (0) | 81 | 1.9 (1.5) | 9 | 1.8 (1.2) | 0 | NA |
| Toaster Pastries | 0 | NA | 0 | NA | 5 | 3 (0) | 6 | 3.2 (0.4) |
| OVERALL | **311** | **2.3 (1.2)** | **3257** | **2.2 (1.4)** | **1845** | **2.3 (1.5)** | **437** | **2.2 (1.7)** |

^1^Marketing power score = the total number of core and broad techniques displayed on the package; ^2^Mulligan et al. (2020). Evaluating the Canadian packaged food supply using Health Canada’s proposed nutrient criteria for restricting food and beverage marketing to children. *IJERPH*, 17(4), 1250

**Appendix S1.** Child-Appealing Packaging (CAP) coding tool

Version April 2021, updated from previous publication (Mulligan et al IJERPH 2021)

*A note on terminology: The terms “child-appealing” or “appealing to children” are used instead of “child-directed”, “child-targeted”, or “marketed to children”, because the latter imply the intent of the manufacturer to direct their product to children. For the purposes of the CAP coding tool, the intent of the manufacturer is not relevant, this tool intends only to determine which products have packaging that may appeal to children and may subsequently be purchased for/by children or consumed by children.*

**Development and purpose**

The child-appealing packaging (CAP) coding tool was developed as a novel methodology to measure the presence, type, and power of child-appealing marketing on product packaging, by evaluating the marketing techniques displayed on the package. The CAP tool was developed based on a published inventory of marketing techniques that have previously been used in child-appealing marketing research[1]. The CAP tool includes marketing techniques that are popular on product packaging specifically (e.g., cartoon characters, toys in the box), as well as techniques that have traditionally been used in other marketing platforms but are now appearing on packaging with evolving marketing practices (e.g., social media handles, scannable codes linked to websites). Marketing techniques included in the CAP tool were categorized into two categories: core techniques and broad techniques, described further below.

**CAP coding tool outcomes**

There are three primary outcome variables of the application of the CAP tool related to the presence, type, and power of child-appealing marketing, detailed in **Table 1.** Briefly, the CAP tool measures the presence of child-appealing marketing (i.e., if the package is child-appealing or not), based on the display of one or more core marketing technique(s), and captures the type of the marketing based on the presence or frequency of display of individual core/broad marketing techniques within the sample. Finally, the CAP tool scores marketing power by summing all the techniques displayed on the package, based on evidence that the number of marketing techniques displayed on the package influences the persuasiveness or intensity of the marketing message[2,3].

**Table 1. Outcome variables of the child-appealing packaging coding tool**

| Outcome Variable | Explanation | Details and Derivation |
| --- | --- | --- |
| Presence of child-appealing marketing | Determines whether the product packaging is child-appealing, based on the display of core marketing techniques. | Binary Variable (i.e., Yes (child-appealing packaging): ≥1 core marketing technique displayed; No (not child-appealing): 0 core techniques displayed) |
| Type of child-appealing marketing | Determines which specific type(s) of core or broad marketing technique(s) are being displayed. | Presence (binary) or frequency (count) of individual core or broad marketing techniques displayed within a sample |
| Power of child-appealing marketing | Determines the power (persuasiveness) of the marketing message based on the number of unique core and broad marketing techniques displayed. | Marketing power score (count variable): sum of all unique core and broad techniques displayed on the package (e.g., 1,2,3, … etc.) |

**Core marketing techniques**

Core techniques are marketing techniques that could independently make a package appealing to children (e.g., cartoon characters or games on the package). Core techniques are also those that are typically included in marketing regulations or restrictions, as these are more ‘objectively’ or defensibly appealing to children.

If a product’s packaging displays one or more of these techniques, then it will be considered to have “child-appealing packaging”. It is important to ensure that that ALL techniques that are displayed are coded (i.e., 1=present/0=absent), as the use of multiple techniques increases the marketing power score.

**Table 2. Core marketing techniques, definitions, and examples**

| # | Technique | Definition | Examples |
| --- | --- | --- | --- |
| 1 | Child-appealing visual/graphical design of package | Intense colors, patterns or visual designs on the packaging or design themes related to fantasy, adventure, magic, sports, etc. that are clearly appealing to children. This can also include the presence of cartoon characters, children, animals, etc. that **are not** branded or licensed characters, celebrities, or tie-ins to child-appealing media.  *Note: this can include child-appealing lettering, if it is enough on its own for the product to be considered “child-appealing”, otherwise code lettering under broad techniques.* | - Space-themed visual design - Rainbow packaging - Chalkboard-style lettering - Cartoon pictures of fictional sports players - Cartoon drawings of animals - Cartoon kids or families |
| 2 | Unconventional shape of the product, featured on the package | The product featured on the packaging has a shape that is unconventional or unusual for that type of product.  E.g. if crackers have a shape other than their usual square or round shape.  *Note: In the case of clear plastic containers where the product is visible through the package, this counts as the shape being visible.* | - Animal shaped crackers - Alphabet shaped pasta - Character, fruit or animal shaped gummies |
| 3 | Unconventional flavour of the product, featured on the package | The product featured on the package has a flavour that is unconventional or unusual for that type of product, or a flavour that is not a ‘real’ or ‘discernable’ flavour.  *Note: this could include the presentation of the flavour in a ‘negative’ way that may appeal to children; e.g., tastes crazy, weird, sour, whacky* | - Tropical Storm Flavour - Cheddarific - Secret Flavour - Chocolate Mud flavour - Cool Cucumber flavour - Raspberry Kiwi Karma |
| 4 | Unconventional colour of the product, featured on the package | The product featured on the package has a colour that is unconventional or unusual for that type of product.  E.g. if crackers are coloured rather than their usual plain/brown colour.  *Note: In the case of clear plastic containers where the product is visible through the package, this counts as the color being visible.* | - Rainbow crackers - Purple Ketchup - Colour changing drink powder - Rainbow fruit roll ups (instead of just red, for example)   *Note: multi-colored candies would NOT be unusual, unless they are described in a more ‘fun’ or child-appealing way.* |
| 5 | Games or activities on package | Presence of games or activities on the package. | - Connect the dots - Mazes - “Count how many snowmen” |
| 6 | Presence of branded characters or spokespersons | Presence of company- or brand-owned characters. | - Tony the Tiger - Toucan Sam - Cap’n Crunch - Kraft Bears - Pillsbury Doughboy |
| 7 | Presence of Licensed Characters | Presence of characters from TV shows, movies, books, etc., that may appeal to children.  Note: human actors, if presented as the character are included here (e.g., Miley Cyrus as Hannah Montana), if portrayed as themselves, include under “Presence of Celebrities” (e.g., Miley Cyrus advertised as Miley Cyrus). | - Dora the Explorer - Batman - Hannah Montana - Star Wars characters |
| 8 | Presence of celebrities | Presence of actors, athletes, musicians, other public figures that may appeal to children | - Derek Jeter - Miley Cyrus |
| 9 | Other child-appealing tie-ins | Other movie/sports/TV show etc. tie-ins that are appealing to children are advertised on the package aside from one of the types of characters or celebrities described above.  *Note: these may appear in addition to the presence of any characters described above* | - NHL tie-ins that feature an ice-rink or hockey equipment with/without a specific player. - Harry Potter tie-in where Hogwarts is presented with/without a character. |
| 10 | Coupons, contests, prizes, or giveaways, specifically appealing to children | Coupons, contests or prizes or giveaways inside the package or to be redeemed later.  *Note: contests or giveaways must be for child-appealing prizes (unlike, for e.g., a Patio Furniture set)* | - Enter to win tickets to a child-appealing movie - Coupon for free yogurt tubes - Toys inside package - Stickers inside package |
| 11 | Appeals to fun or cool | Product packaging makes appeals to the product being fun or funny, having fun while eating the product, being happy, enjoyment, humour, coolness, being cool, etc.  *Note: this includes “fun” packaging (i.e., Packaging that is designed in a way to promote “fun” during eating, or makes eating an “activity”)*  *Note: this could be as part of the product name (e.g., “Fun Dip”, “Kool Kreatures”), if it is clearly “fun/cool” and appealing to children* | - “Have more fun with” - “Feel the bubbles melt” - “Try our crazy/cool new flavors” - “Smiles included” - Display of children having fun, being happy, enjoying the product - Yogurt Tubes - Dunkaroos - Chips Ahoy! - Processed cheese with dipping breadsticks (if “dipping” is promoted as an activity) |
| 12 | Promotion of websites, social media, rewards programs, specifically appealing to children | Product packaging promotes product/brand/company website, child-specific or games-based brand website, social media, or opportunities to “join”, “become a member”, redeem points, and collect rewards or to connect or share with others in a manner that is evidently child-appealing | - “Find more games on [website]” - References to “kids club” or similar |

**Broad marketing techniques**

Broad techniques are marketing techniques that would not on their own cause a product to be considered as “child-appealing”; however, in addition to the core techniques, these could increase the power of the marketing message as a whole. Evidence has shown that marketing techniques such as promoting a product’s health, nutritional or economic value were amongst the most popular techniques used in child-appealing marketing research, despite them not being typical child-appealing techniques[1]. Broad techniques also include marketing techniques that may not appeal directly to children but may appeal to their parents or caregivers (e.g., product benefit claims, convenience packaging), and therefore be purchased for children. These techniques are important to monitor, given that when/if child-appealing marketing is restricted, a proliferation of broad techniques may occur as a means for manufacturers to circumvent regulations and ensure that their products are still consumed by children.

It is important to ensure that ALL techniques that are displayed on the package are coded (i.e., 1=present/0=absent), as the use of multiple techniques increases the marketing power score. Broad techniques should be coded even on products that will NOT ultimately be considered to have child-appealing packaging (i.e., displaying ≥1 core techniques) so that their use can be monitored over time.

**Table 3. Broad marketing techniques, definitions, and examples**

| # | Technique | Definition | Example |
| --- | --- | --- | --- |
| 13 | Interesting font or lettering | Presence of product name or description (e.g., product flavour) written or designed in a colorful, creative, or interesting font that is not on its own enough to make the package “child-appealing”, but may contribute to the overall power of the marketing.  *Note: this broad technique exists due to the difficult nature of identifying child-appealing lettering, and since often products will use bubbly or colorful fonts, but this alone is not always enough to consider a product child-directed.*  *Note: if the lettering is enough to make the product child-appealing, count under technique #1 child-appealing visual/graphic design* | - Aero bar bubble lettering - Corn Pops lettering - Cheetos lettering |
| 14 | Interesting or unconventional product name | Unconventional product name (e.g., strange spelling, rhyming, and alliteration) that may be interesting to children and build marketing power.  *Note: if not counted as part of a core technique (e.g., appeals to fun/cool or visual/graphic design of the package) and* ***not enough*** *to make the product child-appealing on its own.* | - Frooty Hoops - Juicy Jels - Wagon Wheels - “Eat the middle first” |
| 15 | Presence of a logo/image not specifically appealing to children | Presence of a product/brand logo or a cartoon or image that is not specifically appealing to children. This could include pictures of families or children consuming the product (if not in a child-appealing way) | - The man with a moustache in the Pringles logo - Quaker Oats man in the logo - Realistic cows on cheese products - Picture of a child drinking milk on the side of a soy milk package |
| 16 | Promotion of convenient packaging | Specific promotion of the product being packaged in a convenient or easy way.  *Note: if the packaging is promoted as “fun” or as an activity count under “appeals to fun/cool”*  *Note: this does not include single serve packaging (e.g., juice boxes or crackers and cheese packs) without specific promotion of their convenience.* | - “Perfect for on-the-go snacking.” - “Great for packing in lunches” - “Contains 6 easy servings” |
| 17 | Appeals to taste or texture | Product packaging makes appeals to the flavour taste, or texture, of the product, in a way that is not specifically appealing to children.  *Note: this includes if the appeal to taste/texture is part of the product name/brand or product description. | - “New look, same great taste” - “You’ll love it” - “Deep n’ Delicious!” - “Tastes like mama made it” - Promotion of textures (e.g., crunchy, smooth…) - Improved recipe! |
| 18 | Appeals to health or nutrition | Product packaging makes appeals to the healthfulness or nutritional quality of the product, its ability to promote growth, strength, or physical activity. Product packaging displays “healthy foods” alongside the product.  *Note: includes health and nutrition claims/symbols, as well as organic or natural claims/symbols* | - “Helps them grow strong” - “Part of a healthy breakfast” - Fruit featured beside the product on pack (e.g. bowl of strawberries beside cereal) - Source of 5 whole grains - Made with 100%... - Promotion of ‘real’, ‘pure’, ‘natural’ etc. - Gluten free or vegetarian symbol - Peanut free symbol |
| 19 | Appeals to other product benefits | Product packaging makes appeals to other product benefits aside from health/taste/fun. For example, value, quickness, easy preparation, sustainability, philanthropy, etc.  *Note: this does not include small statements (often on the bottom of the package) that the package was made from recycled materials or is recyclable.* | - “Quick and easy” - “Ready in 5 minutes” - “Ready to bake” - Proceeds go to X organization - B Certified Corporations - Promotion of local ingredients - Promotion of “made in Canada” - Value pack/family size/club pack |
| 20 | Recipes | Product packaging displays recipes that can be made using the product  *Note: does not include standard cooking instructions for products that require preparation (e.g., how to cook a frozen pizza) | - Rice crispy squares - Bran muffins - Low calorie smoothies - “Chef’s Tip” |
| 21 | Promotion of websites, social media, rewards programs, not specifically appealing to children | Product packaging promotes product/brand/company website, social media, or opportunities to “join”, “become a member”, redeem points, and collect rewards or to connect or share with others, in a way that is not specifically child-appealing  *Note: does not include link/QR code to company/manufacturer website included as part of contact information on package* | - Social Media links - Links to recipe websites - Links to “create the next flavour of chips” - QR codes (if promoted in a special way) |
| 22 | Coupons, contests, or giveaways, not specifically appealing to children | Coupons, contests or giveaways to be entered or redeemed later that are not specifically appealing to children. | - Tote bags - Access to a free weight loss plan - Patio furniture set |

**References**

1. Mulligan, C.; Kent, M.P.; Christoforou, A.K.; L’Abbé, M.R. Inventory of marketing techniques used in child-appealing food and beverage research: a rapid review. *International journal of public health* **2020**, 1-11.

2. Cao, Z.; Yan, R. Health Creates Wealth? The Use of Nutrition Claims and Firm Financial Performance. *Journal of Public Policy & Marketing* **2016**, *35*, 58-75, doi:10.1509/jppm.14.142.

3. Eisend, M.; Tarrahi, F. The effectiveness of advertising: A meta-meta-analysis of advertising inputs and outcomes. *Journal of Advertising* **2016**, *45*, 519-531.
